# Effect of traditional Chinese medicine on Graves’ disease: a network meta-analysis

**DOI:** 10.3389/fphar.2024.1411459

**Published:** 2024-08-22

**Authors:** Zhuoshi Yang, Na Zhao, Jinchuang Li, Zhouxin Wu, Jian Ma

**Affiliations:** ^1^ First School of Clinical Medicine, Heilongjiang University of Chinese Medicine, Harbin, China; ^2^ Endocrinology Department 1, The First Affiliated Hospital of Heilongjiang University of Traditional Chinese Medicine, Harbin, China

**Keywords:** traditional Chinese medicine, Graves’ disease, network meta-analysis, thyroid stimulating hormone, free triiodothyronine, free thyroxine

## Abstract

**Introduction:** Graves’ disease (GD) is the most common cause of hyperthyroidism and can affect multiple systems of the body. Currently, commonly-used treatment methods for GD have a series of shortcomings. In contrast, traditional Chinese medicine has been proven to be effective in inhibiting the progression of GD and is expected to become a key direction for the development of new drugs in the future. Therefore, a network meta-analysis was performed to compare the impacts of different traditional Chinese medicines on the curative effect, thyroid-stimulating hormone (TSH), free triiodothyronine (FT3), free thyroxine (FT4), thyroglobulin antibody (TGAb), thyroid peroxidase antibody (TPOAb) and thyrotropin receptor antibody (TRAb) in patients with GD.

**Methods:** PubMed, Embase, Cochrane Library, Web of Science, WanFang, Weipu, and CNKI were searched for the randomized controlled trials of traditional Chinese medicine on GD patients up to 19 December 2023. The quality of the included studies was evaluated regarding the risk of bias, and the data were analyzed by R software.

**Results:** Thirty-five articles were included in the analysis, involving 2828 GD patients and traditional Chinese medicines including Bailing Capsule, Jinshuibao Capsule, Astragalus injection, Jiakangling Tablet, Jiakangling Capsule, Tripterygium Wilfordii, Sanjie Xiaoying Decoction, *Prunella vulgaris (L.)* Oral Liquid, *P. vulgaris (L.)* Granules, Xiehuo Xiaoying Recipe, Xiehuo Yangyin Powder, Yikang Pill and Yinjia Pellet. The results of network meta-analysis suggested that for GD patients, Bailing Capsule, Jiakangling Capsule, Tripterygium wilfordii, *P. vulgaris (L.)* Oral Liquid and Yinjia Pellet had better curative effect compared with Western medicine. *Prunella vulgaris (L.)* Granules and Yikang Pill could improve the TSH level. *Prunella vulgaris (L.)* Granules, *P. vulgaris (L.)* Oral Liquid and Yikang Pill could reduce FT3 level. Jiakangling Capsule, *P. vulgaris (L.)* Granules, *P. vulgaris (L.)* Oral Liquid and Yikang Pill could reduce the FT4 level. *Prunella vulgaris (L.)* Oral Liquid can reduce the level of TPOAb and TRAb. Besides, Yinjia Pellet was the most helpful in improving the curative effect. Yikang Pill could best improve TSH. *Prunella vulgaris (L.)* Granules had the best effect on reducing FT3. *Prunella vulgaris (L.)* Granules performed best in reducing FT4. *Prunella vulgaris (L.)* Oral Liquid had the most favorable effect on reducing TPOAb and TRAb.

**Conclusion:** Based on the current research, it is safe to conclude that Chinese medicine can improve the curative effect and TSH level of patients with GD, and reduce the levels of FT3, FT4, TPOAb and TRAb. Besides, Yinjia Pellet is the most helpful in improving the curative effect. Yikang Pill can best improve TSH. *Prunella vulgaris (L.)* Granules have the best effect on reducing FT3. *Prunella vulgaris (L.)* Granules perform best in reducing FT4. *Prunella vulgaris (L.)* Oral Liquid has the most favorable effect on reducing TPOAb and TRAb.

**Systematic Review Registration:** https://www.crd.york.ac.uk/PROSPERO/#recordDetails, identifier CRD42024521912.

## Introduction

Graves’ disease (GD) is an autoimmune thyroid disease, which develops under the influence of immune, genetic, or environmental factors, so that too much thyroid hormone is synthesized and released into the blood, leading to the hyperactivity of digestive, circulatory, and nervous systems. The common symptoms of GD are heat intolerance, excessive sweating, emaciation, emotional lability, and diffuse symmetric goiter. Additionally, most patients have shown exophthalmos condition, which can lead to thyroid complications, hence a life-threatening situation. Graves’ disease development and progression is influenced by heredity, immunity, oxidative stress and inflammatory infiltrate (Antonelli et al., 2020). It affect age groups. According to epidemiological studies, individuals aged between 30 and 60 years are susceptible to GD, that predominantly occurs in women and is the primary cause of hyperthyroidism in iodine-rich areas (Antonelli et al., 2020). Currently, a global incidence of GD is increasing annually, especially in younger age groups. To that, it has become the third high-risk endocrine system disease after glucose metabolism disorder and osteoporosis. In addition, its most common extrathyroid complication is Graves’ ophthalmopathy, which affects about 30% of patients in the late stage of GD (Bartalena and Tanda, 2022). It is believed that the level of thyrotropin receptor antibody (TRAb) in GD patients is increasing, and it will produce pro-inflammatory factors and glycosaminoglycans after combining with thyrotropin receptor (TSHR) in adipocytes and fibroblasts, leading to diplopia, exophthalmos, conjunctival congestion, and even blindness (Bahn, 2015). As influenced by many factors, GD will cause a lot of pressure on patients, and bring a heavy burden to the livelihood of a society. Prophylaxis can reduce its adverse effects on physical and psychological health that become a clinical concern, and more research is needed to resolve this problem.

Apparently, there are three main treatments for Graves’ disease: drug therapy, radioactive iodine therapy and surgical treatment. Drug therapy is preferred in China, but it has a long treatment cycle and a high recurrence rate after drug withdrawal, it may be accompanied by adverse reactions such as allergic skin rash, liver injury and agranulocytosis (Sun et al., 2009; Rivkees and Mattison, 2009; Grzywa et al., 2009). Traditional Chinese medicine has long been used for the treatment of GD and has shown many potential advantages. Clinical practice has revealed that integrating traditional Chinese medicine and Western medicine can not only reduce side effects, but also relieve the clinical symptoms of patients, shorten the medication cycle and reduce the recurrence rate. However, there is no unified standard for the understanding of etiology and pathogenesis of GD in traditional Chinese medicine. The prescriptions and drugs used for treatment differs, while no systematic comparison on efficacy of these treatments in GD. The purpose of this study is to compare the effectiveness of different Chinese medicines in the auxiliary treatment of GD through network meta-analysis, so as to provide a scientific basis for the clinical treatment of GD.

## Methods

### Literature screening

A search as of 19 December 2023 was conducted in the Cochrane, PubMed, Embase, Web of Science, Wan Fang Data, VIP and CNKI databases for Randomized Clinical Trials (RCTs) of traditional Chinese medicine in GD patients. Chinese terms included “Diffuse Toxic Goiter”, “Graves’ Disease”, “Graves’ Disease”, “Traditional Chinese Medicine”, “Traditional Chinese Medical Science”, “Soup”, “Formula”, “Powder”, “Granule”, “Pill”, and “Injection”, while English terms included “Graves Disease” “Disease”, “Graves”, “Basedow Disease”, “Disease, Basedow”, “Graves’ Disease”, “Medicine, Chinese Traditional”, “Traditional Chinese Medicine”, “ChungIHsueh”, “Hsueh, ChungI”, and “Traditional Medicine, Chinese”. The search was carried out by combining subject headings with text-words. The detailed search strategy is provided in Supplementary Table S1.

### Inclusion and exclusion criteria

Inclusion criteria were as follows: Patients met the diagnostic criteria of Graves’ disease (Chinese Guidelines for the Diagnosis, 2007). The experimental group received traditional Chinese medicines (Bailing Capsule, Jinshuibao Capsule, Astragalus injection, Jiakangling Tablet, Jiakangling Capsule, Tripterygium Wilfordii, Sanjie Xiaoying Decoction, *Prunella vulgaris (L.)* Oral Liquid, *P. vulgaris (L.)* Granules, Xiehuo Xiaoying Recipe, Xiehuo Yangyin Powder, Yikang Pill and Yinjia Pellet), whereas the control group received Western medicines. Primary outcomes included thyroid-stimulating hormone (TSH), free triiodothyronine (FT3) and free thyroxine (FT4), while secondary outcomes contained treatment effect, thyroglobulin antibody (TGAb), Thyroid Peroxidase Antibody (TPOAb) and TRAb. RCT were included.

Exclusion criteria were as follows: duplicates, animal experiments, case reports, conference abstracts, reviews, studies with inaccessible full texts, and studies involving participants with other organic diseases.

### Data extraction

Two authors rigorously screened the literature based on predetermined inclusion and exclusion criteria. Any disagreements were resolved through discussion or by seeking a third party’s opinion to reach a consensus. Information extracted from the included studies encompassed key details such as the first author, year of publication, country, sample size, sex, age, interventions, and outcome measures.

### Quality assessment

According to Cochrane Handbook 5.4 for RCT risk of bias assessment, two researchers evaluated the quality of the included studies from seven aspects: random sequence generation, allocation concealment, blinding of patients and investigators, blinding of outcome evaluator, incomplete outcome data, selective reporting and other bias. Studies were rated as “low risk of bias”, “uncertain risk of bias”, or “high bias risk”, accordingly. Any differences in the evaluation results will be handled by a discussion with a third-party researcher to reach a consensus.

### Data analysis

We employed Bayesian network meta-analysis using a prior vague random effects model with the R 4.3.2 software (R Foundation for Statistical Computing), and adopted Markov Chain Monte Carlo methods (Jansen et al., 2008) to obtain the best pooled estimate and probabilities of each treatment regimen. Continuous outcomes were presented as the posterior mean difference (MD) and its 95% confidence interval (CI). Binary outcomes were expressed by odds ratio (OR) and its 95% confidence interval (CI). The surface under the cumulative ranking curve (SUCRA) percentages were calculated to estimate the probability of each intervention being the most effective. Network plots and funnel plots were generated using STATASE -64. In the network plots, each node represented a medication, and the edges represented the available comparisons. The size of each node was proportional to the number of patients included. Cumulative probability plots were created using the ggplot2 package.

## Results

**FIGURE 1:**
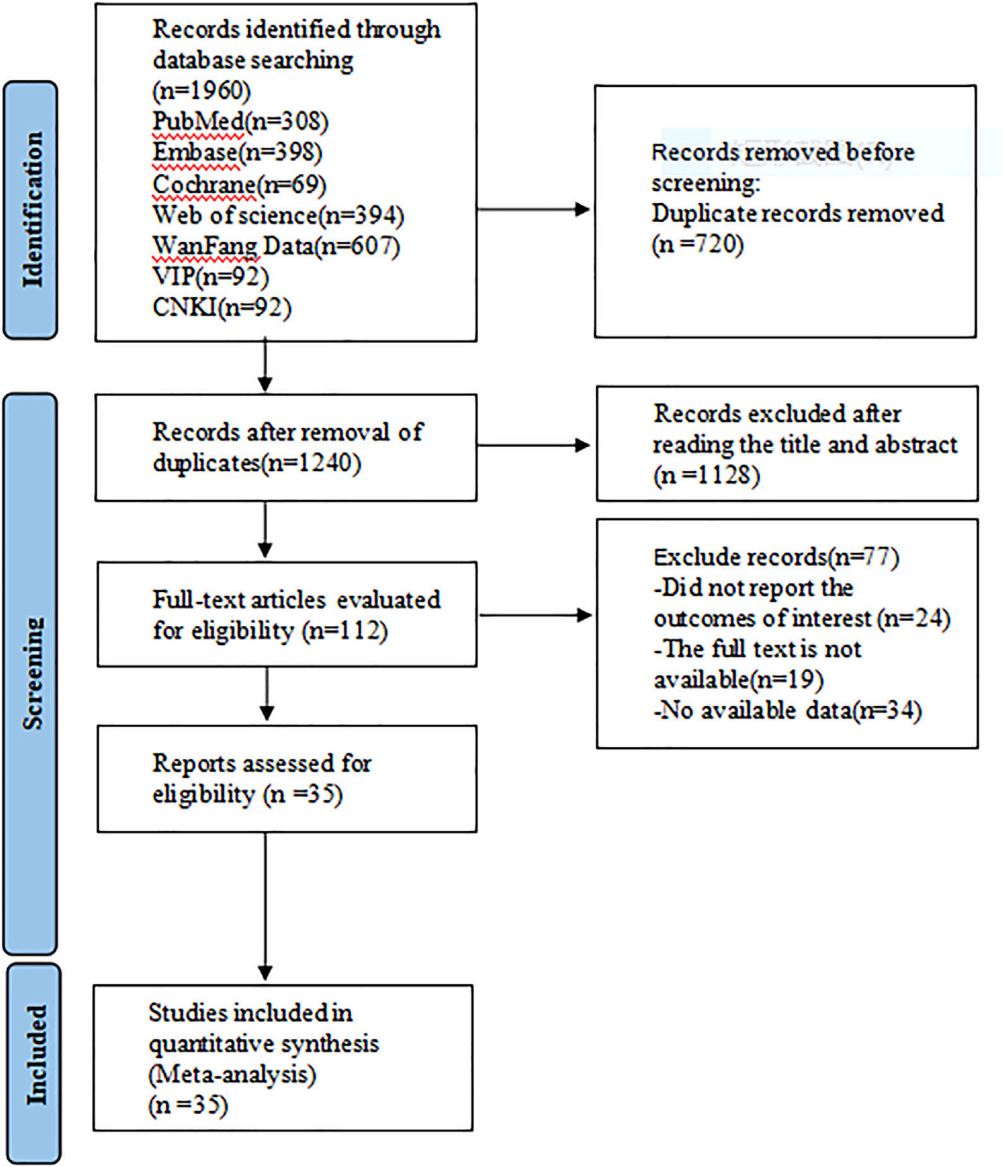
Literature retrieval flow chart.

A preliminary search of the databases yielded 1960 articles. After the removal of 720 duplicates, 1128 articles were excluded based on title and abstract screening, and 77 more were excluded after full-text reading. Ultimately, 35 (Meng et al., 2019; Haiyan, 2019; Changlin, 2010; Hongjun, 2020; Yidan, 2021; Zhaoli and Xiaobin, 2021; Ming et al., 2014; Hansong et al., 2011a; Shengli, 2009; Xiangming and Wenchun, 2003; Hongyao, 2009; Fengling, 2006; Wen, 2018; Minhong and Zhihong, 2016; Ping, 2009; Zhonghua and Hongjuan, 2003; Zhongyu, 2020; Yaowu, 2017; Min et al., 2019; Lichao et al., 2020; Yi and Xuewei, 2023; Huilin et al., 2020; Yongqing et al., 2017; Shengben, 2012; Yaowu, 2016; Xiao et al., 2018; Hui et al., 2022; Xuemei et al., 2003; Hansong et al., 2011b; Shuguang et al., 2010; Yuming et al., 2003; Yujuan et al., 2015; Zhenbo and Shufang, 2003; Tonghuan and Fangfang, 2008; Xinyi et al., 2021) articles were included in the analysis (Figure 1).

### Basic characteristics and risk of bias assessment of the literature

**FIGURE 2:**
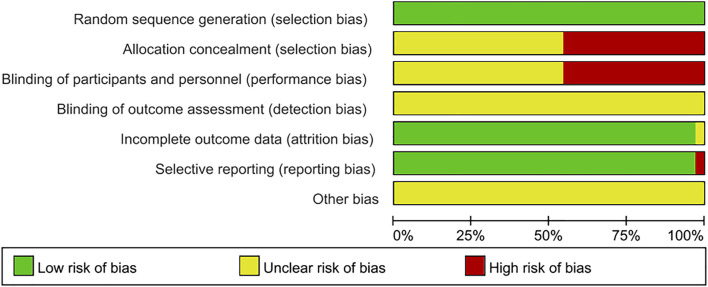
Risk of bias graph.

**FIGURE 3:**
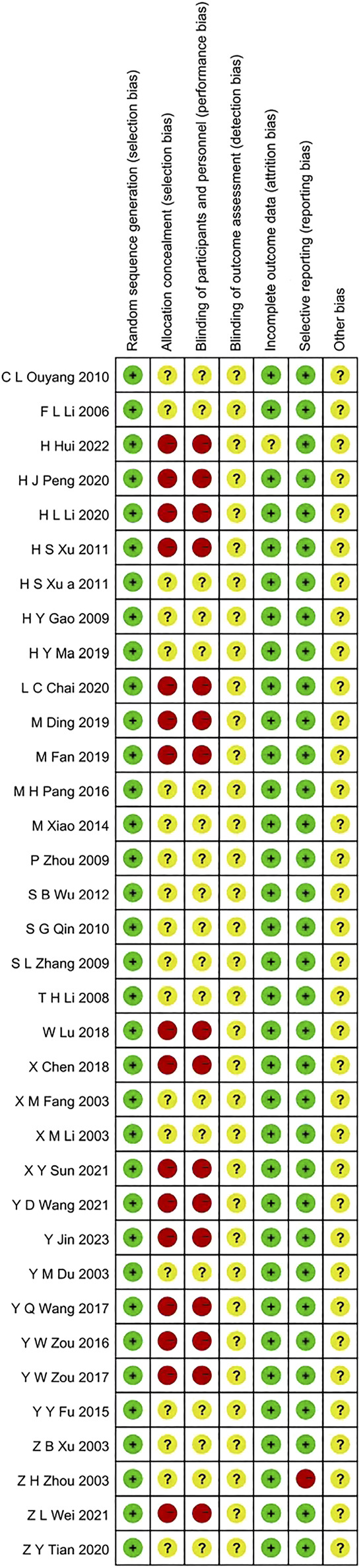
Risk of bias summary.

In these 35 articles, there were 2828 GD patients and traditional Chinese medicines including Bailing Capsule, Jinshuibao Capsule, Astragalus injection, Jiakangling Tablet, Jiakangling Capsule, Tripterygium Wilfordii, Sanjie Xiaoying Decoction, *P. vulgaris (L.)* Oral Liquid, *P. vulgaris (L.)* Granules, Xiehuo Xiaoying Recipe, Xiehuo Yangyin Powder, Yikang Pill and Yinjia Pellet in different doses (Table 1). All included studies explicitly described their blinding methods. The main risk of bias arose from deviations from the intended interventions, as assessed in the risk of bias summary (Figures 2, 3).

**TABLE 1:** Characteristics of the included studies.

Study	Year	Sample size	Gender (M/F)	Mean age (years)	Intervention	Outcome
M Ding	2019	BLC + WM:42 WM:42	62/22	BLC + WM:35.25 WM:34.98	BLC:2 g, thrice/day WM:MMI	F5; F6; F7; F12; F17; F29; F30; F31; F59; F66; F67; F68; F69
H Y Ma	2019	BLC + WM:30 WM:29	33/26	BLC + WM:35.9 WM:36.3	BLC:2 g, thrice/day WM:PTU	F1; F12; F17
C L Ouyang	2010	BLC + WM:24 WM:26	11/39	BLC + WM:41 WM:40	BLC:1 g, thrice/day WM:MMI	F5; F6; F7; F17; F23; F32
H J Peng	2020	BLC + WM:37 WM:37	40/34	BLC + WM:33.61 WM:33.52	BLC:2 g, thrice/day WM:MMI	F1; F12; F17
Y D Wang	2021	BLC + WM:40 WM:40	32/48	BLC + WM:35.67 WM:35.33	BLC:2 g, thrice/day WM:MMI	F5; F6; F7; F12; F17
Z L Wei	2021	BLC + WM:49 WM:48	41/56	BLC + WM:36.25 WM:36.42	BLC:2 g, thrice/day WM:prednisolone	F1; F11; F12; F17; F29; F30; F31
M Xiao	2014	JSBC + WM:30 WM:28	23/35	JSBC + WM:33 WM:31	JSBC:3pill, thrice/day WM:MMI	F3; F4; F5; F6; F7; F17
H S Xu	2011	BLC + WM:32 WM:28	16/44	BLC + WM:31.5 WM:32.6	BLC:2 g, thrice/day WM:PTU	F5; F6; F7; F12; F17
S L Zhang	2009	BLC + WM:40 WM:40	21/59	BLC + WM:33.2 WM:34.3	BLC:10pill, thrice/day WM:PTU	F1; F3; F4; F5; F6; F7; F23; F25; F26
X M Fang	2003	HQI + WM:41 WM:41		HQI + WM:32 WM:32	HQI:60 mL, once/day WM:MMI	F5; F6; F7; F11; F27; F44; F45; F46; F47
H Y Gao	2009	HQI + WM:22 WM:21	17/26	HQI + WM:28.5 WM:30.5	HQI:60 mL, once/day WM:MMI	F1; F3; F4; F5; F6; F7; F23
F L Li	2006	JKLT + WM:48 WM:52	21/79	JKLT + WM:33.7 WM:34.27	JKLT:3pill, thrice/day WM:MMI	F2; F6; F7; F23; F24
W Lu	2018	JKLC + WM:67 WM:67	47/87	JKLC + WM:44.05 WM:43.11	JKLC:4pill, thrice/day WM:PTU	F1; F2; F5; F6; F7; F11; F12; F23
M H Pang	2016	JKLT:+WM:30 WM:30	12/48	JKLT:+WM:25.8 WM:25.8	JKLT:6∼7pill, thrice/day WM:^131^I	F5; F6; F7; F17; F23; F34
P Zhou	2009	TW + WM:34 WM:23	14/43	TW + WM:35.9 WM:36.71	TW:10pill, thrice/day WM:PTU	F1; F3; F4; F5; F6; F7; F23; F25; F26
Z H Zhou	2003	TW + WM:30 WM:30	9/51	TW + WM:31.2 WM:34.1	TW:1 mg·kg^-1^·d ^-1^ WM:MMI	F5; F6; F7; F23; F32
Z Y Tian	2020	SJXYD + WM:45 WM:45	18/72	SJXYD + WM:34.02 WM:32.93	SJXYD:100 mL, twice/day WM:MMI	F1; F5; F6; F7; F17; F24
Y W Zou	2017	SJXYD + WM:45 WM:45	20/70	SJXYD + WM:38.5 WM:37.8	SJXYD:1Sachet, twice/day WM:PTU, MMI	F1; F2; F5; F6; F7; F17
M Fan	2019	XKCOL + WM:96 WM:96	50/142	XKCOL + WM:37 WM:36.5	XKCOL:10 mL, twice/day WM:^131^I	F1; F5; F6; F7; F11; F12; F17; F24
L C Chai	2020	XKCOL + WM:50 WM:50	33/67	XKCOL + WM:48 27 WM:45 83	XKCOL:10 mL, twice/day WM:MMI	F1; F2; F3; F4; F5; F6; F7; F11; F12; F17; F23; F59
Y Jin	2023	XKCG + WM:39 WM:39	28/50	XKCG + WM:42.35 WM:41.2	XKCG:2 g, twice/day WM:MMI	F1; F5; F6; F7; F17; F43
H L Li	2020	XKCOL + WM:40 WM:40	25/55	XKCOL + WM:41.67 WM:42.59	XKCOL:10 mL, twice/day WM:^131^I	F1; F5; F6; F7; F11; F12; F23; F27; F34; F35
Y Q Wang	2017	XKCG + WM:41 WM:39	25/55	XKCG + WM:38 WM:38	XKCG:9 g, twice/day WM:MMI	F5; F6; F7; F11; F12; F13; F14; F27; F34; F35
S B Wu	2012	XKCOL + WM:60 WM:60	34/86	XKCOL + WM:32.16 WM:31.52	XKCOL:10 mL, twice/day WM:MMI	F1; F2; F5; F6; F7
Y W Zou	2016	XKCOL + WM:45 WM:45	20/70	XKCOL + WM:38.5 WM:37.8	XKCOL:10 mL,twice/day WM:PTU, MMI	F1; F2; F5; F6; F7; F17
X Chen	2018	XHXYP + WM:30 WM:30	13/47	XHXYP + WM:37.73 WM:34.35	XHXYP:1sachet, twice/day WM:MMI	F5; F6; F7; F17; F23; F24
H Huang	2022	XHXYP + WM:40 WM:40	13/55	XHXYP + WM:37.5 WM:31	XHXYP:1sachet, twice/day WM:MMI	F2; F5; F6; F7; F17; F23; F28; F36
X M Li	2003	XHYYP + WM:30 WM:30	17/43	XHYYP + WM:39.38 WM:40.27	XHYYP:10 g, twice/day WM:MMI	F1; F3; F4; F5; F23; F24; F28
H S Xu a	2011	XHYYP + WM:25 WM:25	11/39	XHYYP + WM:33.26 WM:31.23	XHYYP:10 g, twice/day WM:MMI	F5; F6; F7; F13; F39; F40
S G Qin	2010	YKP + WM:63 WM:63	29/97	YKP + WM:38.6 WM:38.6	YKP:4.7 g, twice/day WM:MMI	F2; F11; F12; F16; F24
Y M Du	2003	YKP + WM:31 WM:25	19/37	YKP + WM:20.23 WM:20.23	YKP:1pill, twice/day WM:ATD	F5; F6; F7; F11; F12; F27
Y J Fu	2015	YKP + WM:55 WM:55	31/79	YKP + WM:41.2 WM:40.8	YKP:7 5 g, twice/day WM:^131^I	F1; F3; F4; F5; F6; F7; F11; F12; F27; F50; F51; F52; F53
Z B Xu	2003	YKP + WM:35 WM:25	8/52	YKP + WM:29.8 WM:31.14	YKP:1pill, twice/day WM:PTU + Propranolol	F2; F6; F7; F16
T H Li	2008	YJP + WM:30 WM:30	14/46	YJP + WM:35.7 WM:35.99	YJP:1sachet WM:MMI	F1; F5; F6; F7; F23; F24
X Y Sun	2021	YJP + WM:40 WM:40	29/51	YJP + WM:34.8 WM:37.58	YJP:3 g, twice/day WM:MMI, PTU	F1; F2; F3; F4; F5; F6; F7; F16; F17; F23; F24; F59

M/F, Male/female; WM, Western medicine; MMI, Methimazole; PTU, Propylthiouracil; ATD, Antithyroid drug; BLC, Bailing Capsule; JSBC, Jinshuibao Capsule; HQI, Huangqi Injection; JKLT, Jiakangling Tablet; JKLC, Jiakangling Capsule; TW, Tripterygium wilfordii; SJXYD, Sanjie Xiaoying decoction; XKCOL, Xiakucao oral liquid; XKCG, Xiakucao granule; XHXYF, Xiehuo Xiaoying formula; XHYYP, Xiehuo Yangying powder; YKP, Yikang pill; YJP, Yinjia pill; F1, Efficacy; F2, Improvement of thyroid enlargement; F3:TT3; F4:TT4; F5:TSH; F6:FT3; F7:FT4; F11:TgAb; F12:TPOAb; F13:IFN-γ; F14:IL-10; F16:Improvement of exophthalmos; F17:TRAb; F23:Incidence of adverse reactions; F24:Comparison of syndrome scores; F25:Heart rate; F26:Weight; F27:TMAb; F28:Dosage of methimazole; F29:CD4+; F30:CD8+; F31:CD4+/CD8+; F32:TSAb; F34:TNG-α; F35:IL-6; F36:PSV; F39:IL-4; F40:IFN-γ/IL-4; F43:FGF21; F44:CD3; F45:CD4; F46:CD8; F47:CD4/CD8; F50:FFA; F51:APN; F52:ADSF; F53:LP; F59:Recurrence rate; F66:CysC; F67:TLR4; F68:NF-κB; F69:TGF-β1.

### Results of the network meta-analysis

#### Curative effect

**FIGURE 4:**
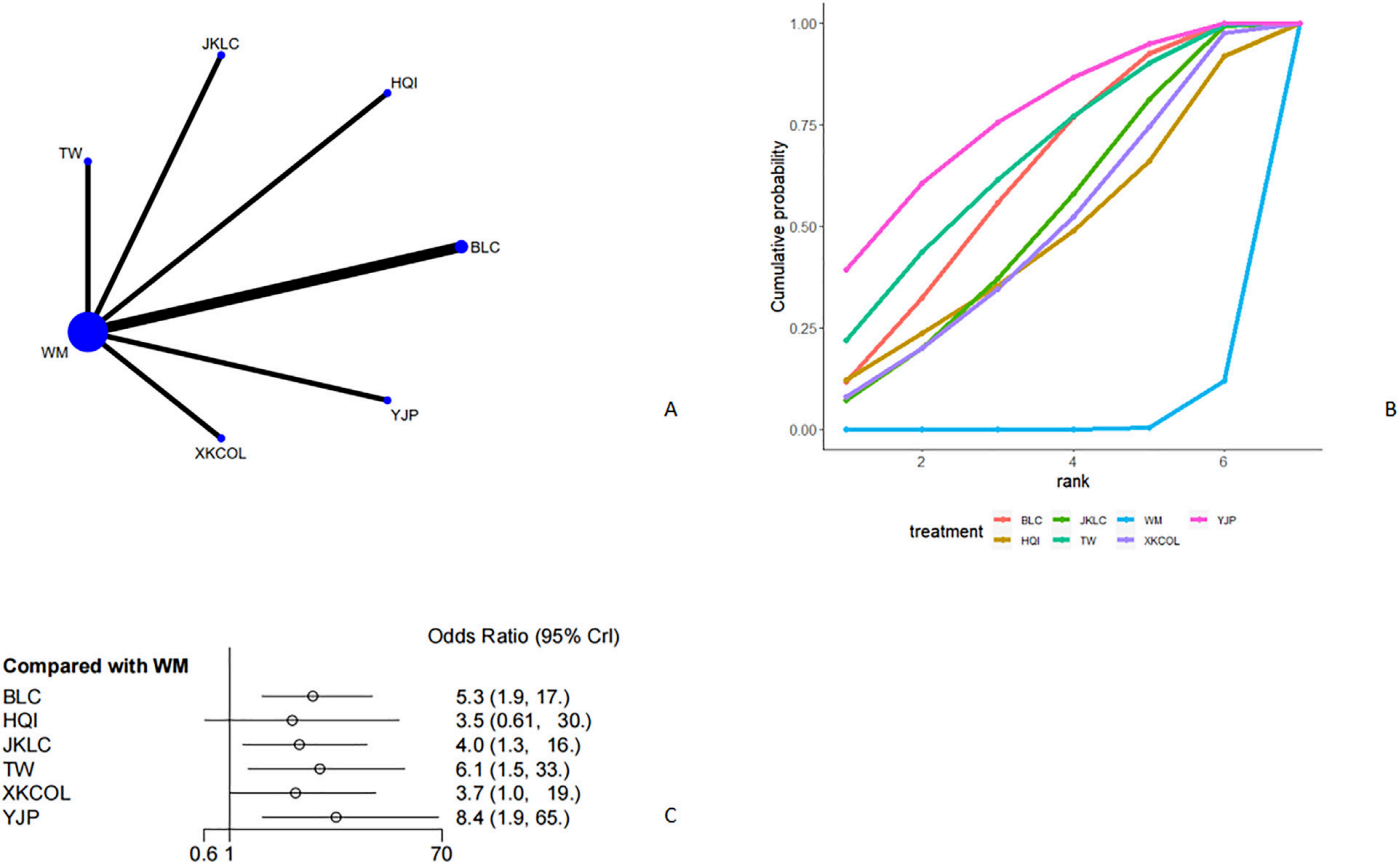
Meta analysis of therapeutic effect (**(A)**: Network plot, **(B)** Area under the cumulative probability curve, **(C)** forest plot).

Seven studies [11, 18, 20, 22, 24, 29, 44] discussed the curative effect (Figure 4, Figure 4A). From the network plot, we found no closed loop, and there was a direct comparison of Western medicine with Tripterygium Wilfordii, Jiakangling Capsule, Astragalus injection, Bailing Capsule, Yinjia Pellet, and *P. vulgaris (L.)* Oral Liquid. Compared with Western medicine, Bailing capsule [OR = 5.3, 95%CI (1.9,17)], Jiakangling Capsule [OR = 4.0, 95%CI (1.3,16)], Tripterygium Wilfordii [OR = 6.1, 95% CI (1.5,33)], *P. vulgaris (L.)* Oral Liquid [OR = 3.7, 95%CI (1.0,19)], and Yinjia Pellet [OR = 8.4, 95%CI (1.9,65)] could improve the curative effect of GD patients (Figure 4C), and there was no significant difference (Supplementary Table S2). According to the SUCRA, Yinjia Pellet ranked the highest (76.4%), followed by Tripterygium Wilfordii (65.6%), Bailing Capsule (61.3%) and Western medicine (2%) (Figure 4B; Table 2).

**TABLE 2:** Comprehensive ranking of SUCRA.

	Efficacy (%)	TSH(%)	FT_3_ (%)	FT_4_ (%)	TGAb (%)	TPOAb (%)	TRAb (%)
BLC	61.3	36	33.3	31	47	34.2	56.5
HQI	46.1	28.9	64.8	37.6	38.5		
JKLC	50.6	40	80.8	84	49.6	38.4	
JKLT		73.1	59.2	47.6			41.1
JSBC		39.3	35.3	22			44.2
SJXYD		36.4	52.8	59.7			67.7
TW	65.6	62.3	49.5	58.3			
WM	2	19.4	15.7	20.3	30.9	7.35	22.1
XHXYP		73	24.7	25.2			32.6
XHYYP		15.7	36.4	50.4			
XKCG		78.8	84.3	84.7	39.6	76.4	65.1
XKCOL	47.9	53.4	61.1	67.3	74.3	85.3	84.1
YJP	76.4	50.8	34.1	37.4			36.5
YKP		92.8	67.9	74.5	70.2	58.2	

#### TSH

**FIGURE 5:**
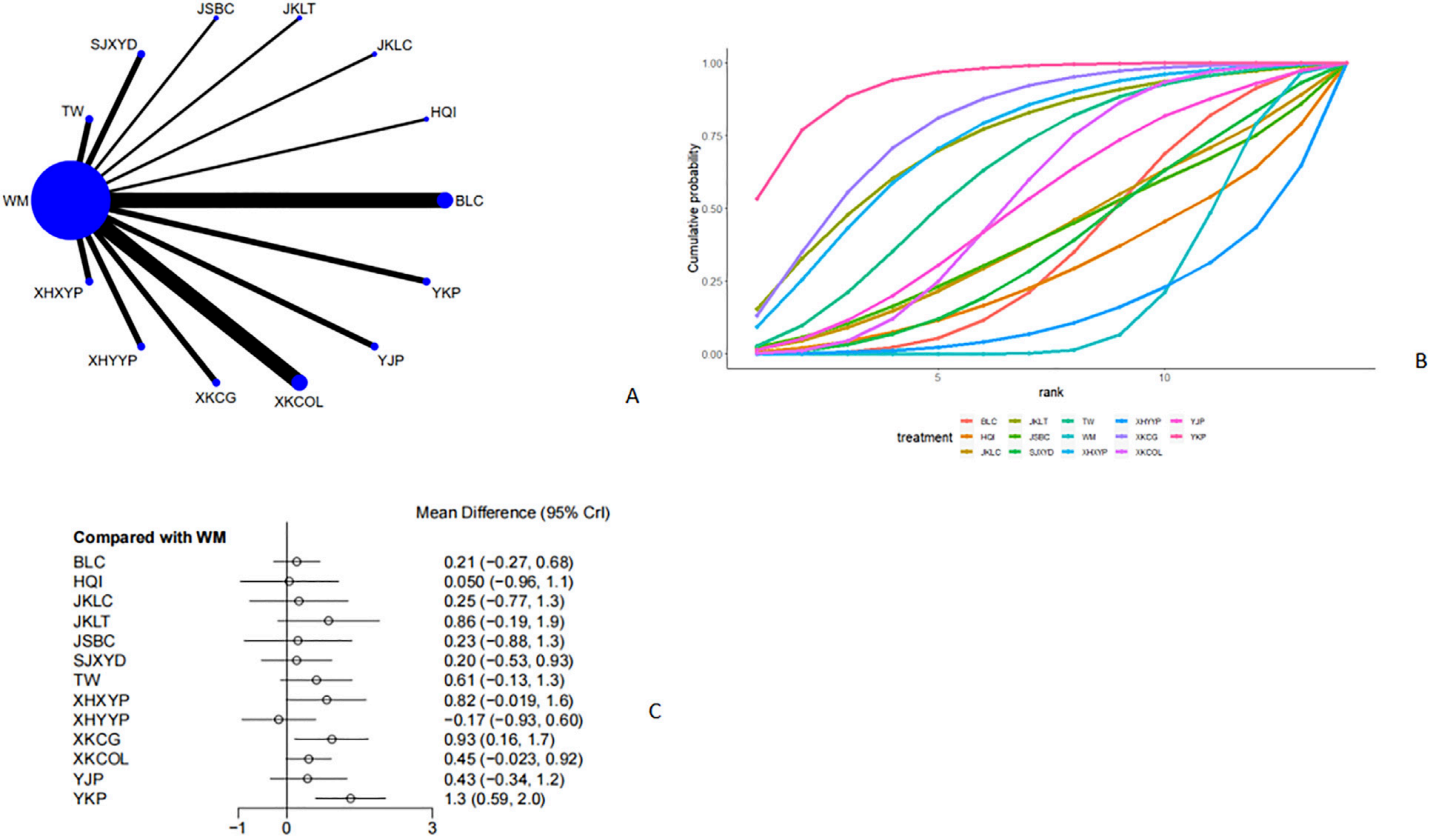
Meta analysis of TSH (**(A)**: Network plot, **(B)** Area under the cumulative probability curve, **(C)** forest plot).

Twenty-eight studies (Meng et al., 2019; Changlin, 2010; Yidan, 2021; Ming et al., 2014; Hansong et al., 2011a; Shengli, 2009; Xiangming and Wenchun, 2003; Wen, 2018; Minhong and Zhihong, 2016; Ping, 2009; Zhonghua and Hongjuan, 2003; Zhongyu, 2020; Yaowu, 2017; Min et al., 2019; Lichao et al., 2020; Yi and Xuewei, 2023; Huilin et al., 2020; Yongqing et al., 2017; Shengben, 2012; Yaowu, 2016; Xiao et al., 2018; Hui et al., 2022; Xuemei et al., 2003; Hansong et al., 2011b; Yuming et al., 2003; Yujuan et al., 2015; Tonghuan and Fangfang, 2008; Xinyi et al., 2021) reported TSH (Figure 5, Figure 5A). From the network plot, no closed loop was found, and there was a direct comparison of Western medicine with Tripterygium Wilfordii, Sanjie Xiaoying Decoction, Jinshuibao Capsule, Jiakangling Tablet, Astragalus injection, Bailing Capsule, Yikang Pill, Jiakangling Capsule, Yinjia Pellet, *P. vulgaris (L.)* Oral Liquid, *P. vulgaris (L.)* Granules, Xiehuo Yangyin Powder, and Xiehuo Xiaoying Recipe. Compared with Western medicine, *P. vulgaris (L.)* Granules [MD = 0.93, 95%CI (0.16,1.7)] and Yikang Pill [MD = 1.3, 95%CI (0.59,2.0)] could improve the TSH level of GD patients (Figure 5C), and there was no significant difference (Supplementary Table S3). According to the SUCRA curve, Yikang Pill ranked the highest (92.8%), followed by *P. vulgaris (L.)* Granules (78.8%), Jiakangling Tablet (73.1%) and Xiehuo Yangyin Powder (15.7%) (Figure 5B; Table 2).

#### FT3

**FIGURE 6:**
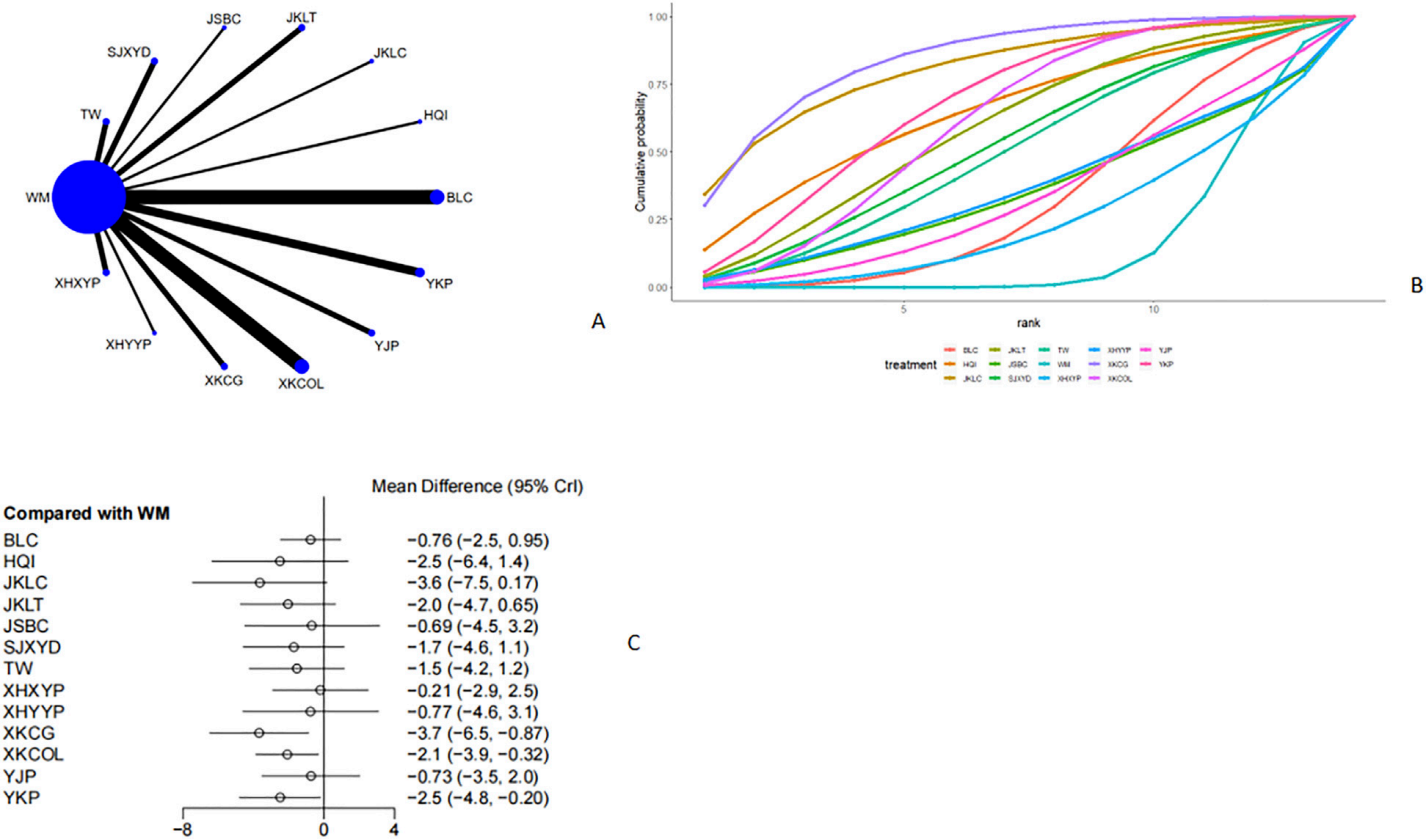
Meta analysis of FT3 (**(A)**: Network plot, **(B)** Area under the cumulative probability curve, **(C)** forest plot).

Twenty-nine studies (Meng et al., 2019; Changlin, 2010; Yidan, 2021; Ming et al., 2014; Hansong et al., 2011a; Shengli, 2009; Xiangming and Wenchun, 2003; Fengling, 2006; Wen, 2018; Minhong and Zhihong, 2016; Ping, 2009; Zhonghua and Hongjuan, 2003; Zhongyu, 2020; Yaowu, 2017; Min et al., 2019; Lichao et al., 2020; Yi and Xuewei, 2023; Huilin et al., 2020; Yongqing et al., 2017; Shengben, 2012; Yaowu, 2016; Xiao et al., 2018; Hui et al., 2022; Hansong et al., 2011b; Yuming et al., 2003; Yujuan et al., 2015; Zhenbo and Shufang, 2003; Tonghuan and Fangfang, 2008; Xinyi et al., 2021) mentioned FT3 (Figure 6, Figure 6A). From the network plot, it was found that no closed loop was formed, and there was a direct comparison of Western medicine with Tripterygium Wilfordii, Sanjie Xiaoying Decoction, Jinshuibao Capsule, Jiakangling Tablet, Jiakangling Capsule, Astragalus injection, Bailing Capsule, Yikang Pill, Yinjia Pellet, *P. vulgaris (L.)* Oral Liquid, *P. vulgaris (L.)* Granules, Xiehuo Yangyin Powder, and Xiehuo Xiaoying Recipe. Compared with Western medicine, *P. vulgaris (L.)* Granules [MD = −3.7, 95%CI (−6.5, −0.87)], *P. vulgaris (L.)* Oral Liquid [MD = −2.1, 95%CI (−3.9, −0.32)] andYikang Pill [MD = −2.5, 95%CI (−4.8, −0.2)] could reduce FT3 level in GD patients (Figure 6C), but there was no significant difference (Supplementary Table S4). According to the SUCRA curve, *P. vulgaris (L.)* Granules ranked the highest (84.3%), followed by Jiakangling Capsule (80.8%), Yikang Pill (67.9%) and Western medicine (15.7%) (Figure 6B; Table 2).

#### FT4

**FIGURE 7:**
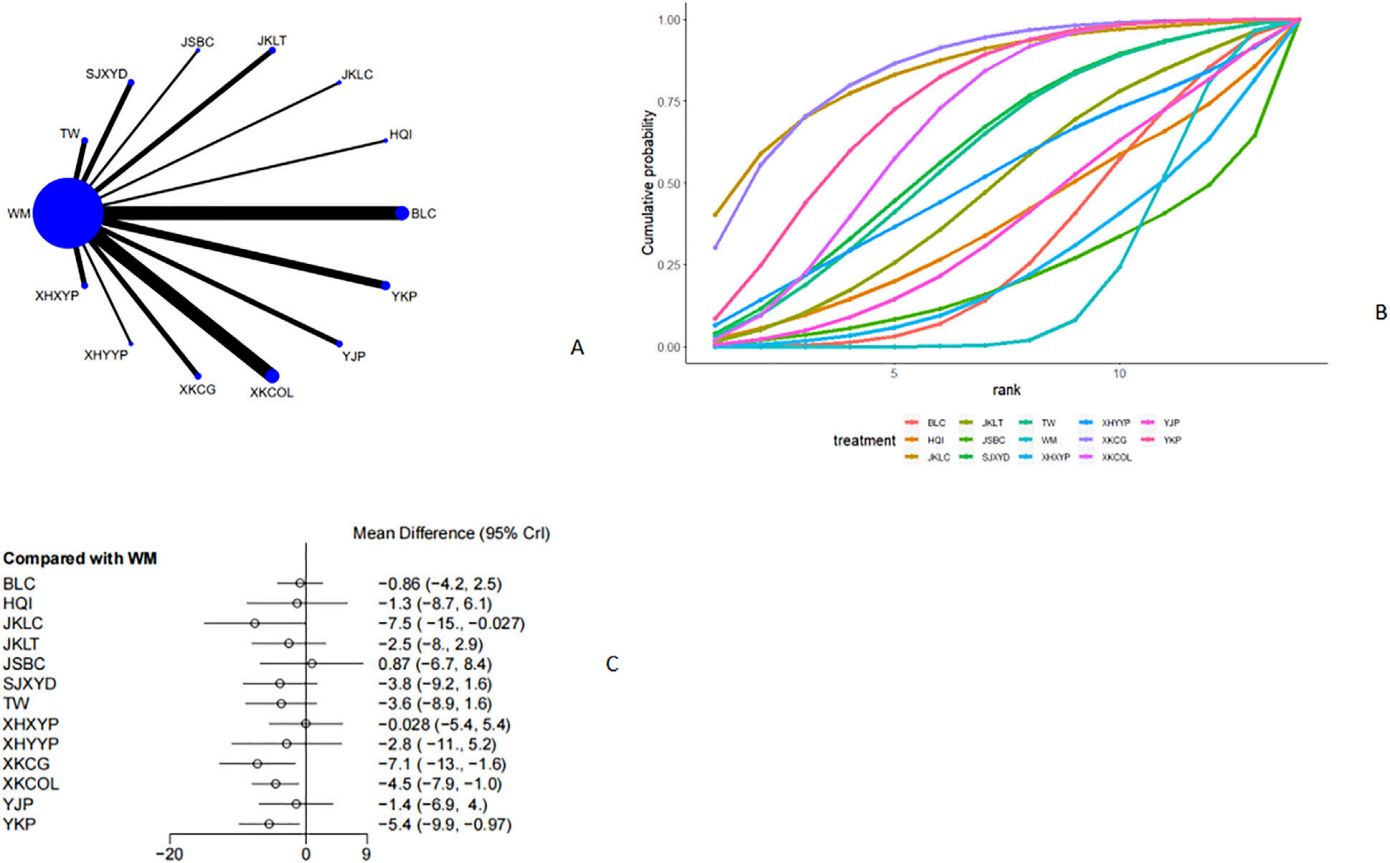
Meta analysis of FT4 (**(A)**: Network plot, **(B)** Area under the cumulative probability curve, **(C)** forest plot).

Twenty-nine studies (Meng et al., 2019; Changlin, 2010; Yidan, 2021; Ming et al., 2014; Hansong et al., 2011a; Shengli, 2009; Xiangming and Wenchun, 2003; Fengling, 2006; Wen, 2018; Minhong and Zhihong, 2016; Ping, 2009; Zhonghua and Hongjuan, 2003; Zhongyu, 2020; Yaowu, 2017; Min et al., 2019; Lichao et al., 2020; Yi and Xuewei, 2023; Huilin et al., 2020; Yongqing et al., 2017; Shengben, 2012; Yaowu, 2016; Xiao et al., 2018; Hui et al., 2022; Hansong et al., 2011b; Yuming et al., 2003; Yujuan et al., 2015; Zhenbo and Shufang, 2003; Tonghuan and Fangfang, 2008; Xinyi et al., 2021) reported FT4, as shown in Figure 7 and Figure 7A. From the network plot, we found no closed loop, and there was a direct comparison of Western medicine with Tripterygium Wilfordii, Sanjie Xiaoying Decoction, Jinshuibao Capsule, Jiakangling Tablet, Jiakangling Capsule, Astragalus injection, Bailing Capsule, Yikang Pill, Yinjia Pellet, *P. vulgaris (L.)* Oral Liquid, *P. vulgaris (L.)* Granules, Xiehuo Yangyin Powder, and Xiehuo Xiaoying Recipe. Compared with Western medicine, Jiakangling Capsule [MD = −7.5, 95%CI (−15, −0.027)], *P. vulgaris (L.)* Granules [MD = −7.1, 95%CI (−13, −1.6)] and *P. vulgaris (L.)* Oral Liquid [MD = -4.5, 95%CI (−7.9,-1.0)] and Yikang Pill [MD = −5.4,95%CI (−9.9, −0.97)] could reduce the FT4 level of GD patients (Figure 7C), and no significant difference was observed (Supplementary Table S5). According to the SUCRA curve, *P. vulgaris (L.)* Granules ranked the highest (84.7%), followed by Jiakangling Capsule (84%), Yikang Pill (74.5%) and Western medicine (20.3%) (Figure 7B; Table 2).

#### TGAb

**FIGURE 8:**
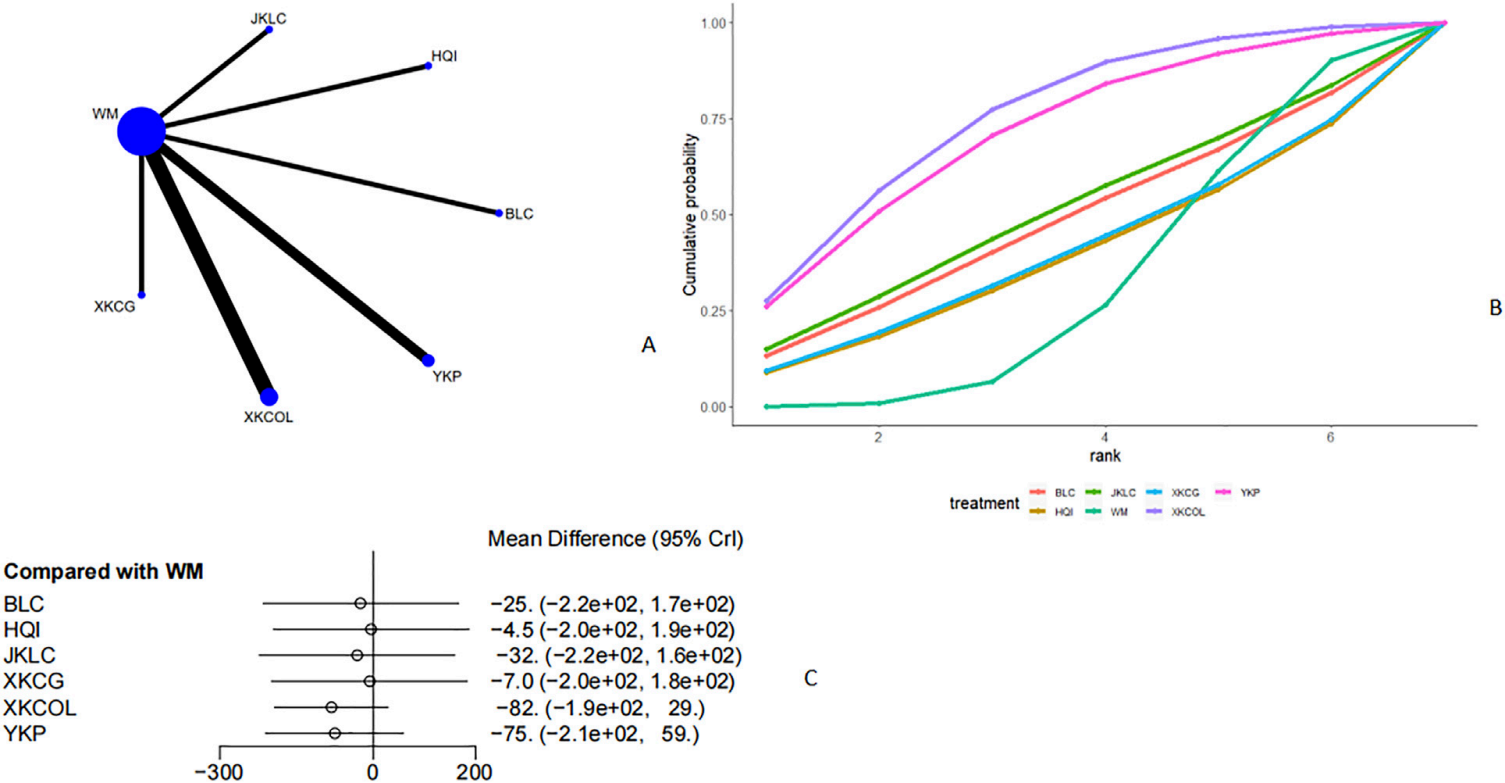
Meta analysis of TGAb (**(A)**: Network plot, **(B)** Area under the cumulative probability curve, **(C)** forest plot).

Nine studies (Zhaoli and Xiaobin, 2021; Xiangming and Wenchun, 2003; Wen, 2018; Min et al., 2019; Lichao et al., 2020; Huilin et al., 2020; Yongqing et al., 2017; Shuguang et al., 2010; Yujuan et al., 2015) reported TGAb (Figure 8, Figure 8A). No closed loop was observed from the network plot, and it was found that there was a direct comparison of Western medicine with Jiakangling Capsule, Astragalus injection, Bailing Capsule, Yikang Pill, *P. vulgaris (L.)* Oral Liquid, and *P. vulgaris (L.)* Granules. There was no significant difference between Western medicine and Chinese medicines in reducing TGAb (Figure 8C). According to the league table, the difference of variable Chinese medicines in reducing TGAb was not significant (Supplementary Table S6). According to the SUCRA curve, *P. vulgaris (L.)* Oral Liquid ranked the highest (74.3%), followed by Yikang Pill (70.2%), Jiakangling Capsule (49.6%) and Western medicine (30.9%) (Figure 8B; Table 2).

#### TPOAb

**FIGURE 9:**
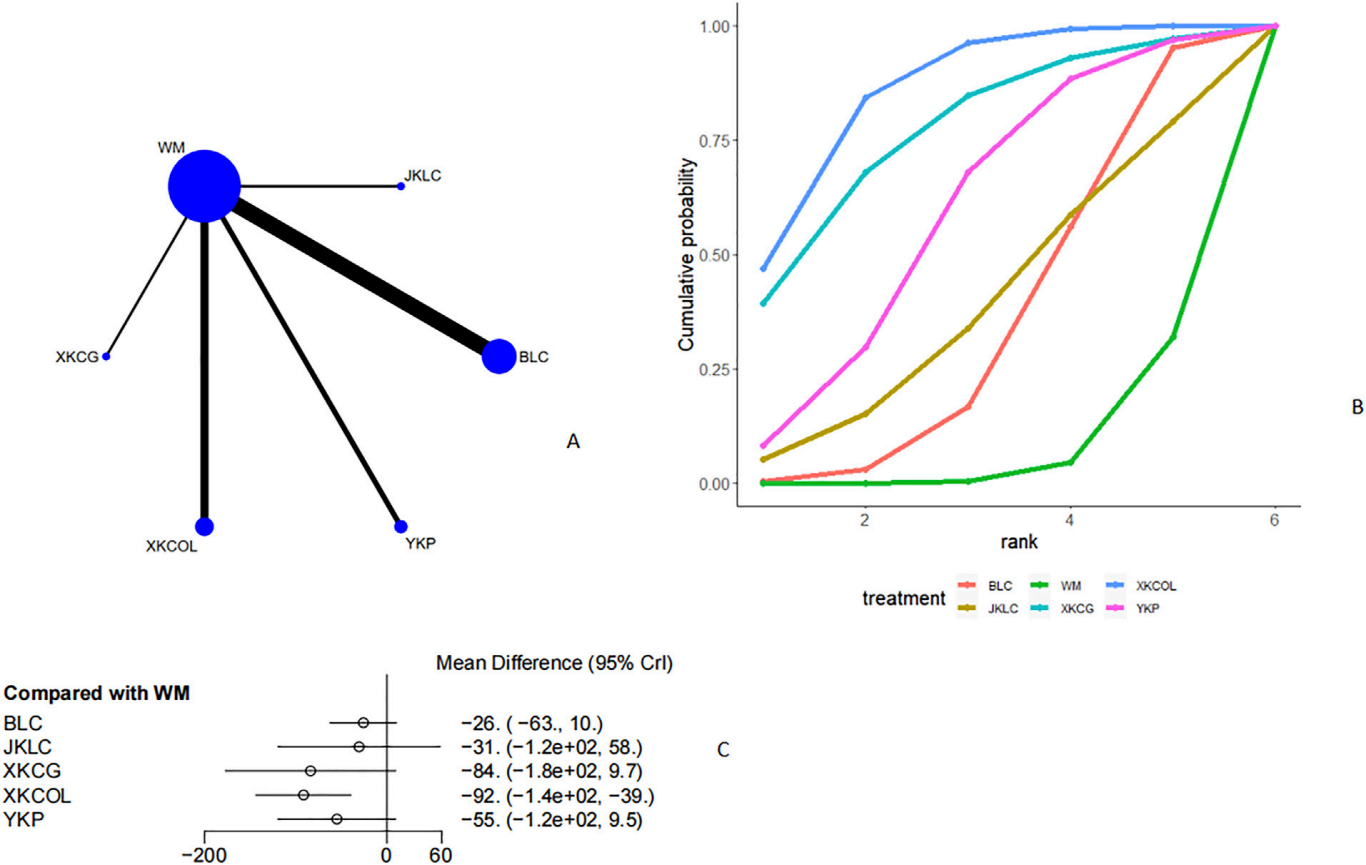
Meta analysis of TPOAb (**(A)**: Network plot, **(B)** Area under the cumulative probability curve, **(C)** forest plot).

Thirteen studies (Meng et al., 2019; Haiyan, 2019; Hongjun, 2020; Yidan, 2021; Zhaoli and Xiaobin, 2021; Hansong et al., 2011a; Wen, 2018; Min et al., 2019; Lichao et al., 2020; Huilin et al., 2020; Yongqing et al., 2017; Shuguang et al., 2010; Yujuan et al., 2015) discussed TPOAb (Figure 9, Figure 9A). From the network plot, no closed loop was formed, and it could be seen that there was a direct comparison of Western medicine with Jiakangling Capsule, Bailing Capsule, Yikang Pill, *P. vulgaris (L.)* Oral Liquid, and *P. vulgaris (L.)* Granules. Compared with Western medicine, *P. vulgaris (L.)* oral liquid [MD = −92, 95%CI (−1.4e+02, −39)] could reduce the level of TPOAb in GD patients (Figure 9C). From the league table, it could be seen that *P. vulgaris (L.)* Oral Liquid [MD = -92,95% CI (−1.4e+02,39)] could better reduce TPOAb level than Bailing Capsule (Supplementary Table S7), and ranked the highest in the SUCRA curve (85.3%), followed by *P. vulgaris (L.)* Granules (76.4%), Yikang Pill (58.2%), and Western medicine (7.35%) (Figure 9B; Table 2).

#### TRAb

**FIGURE 10:**
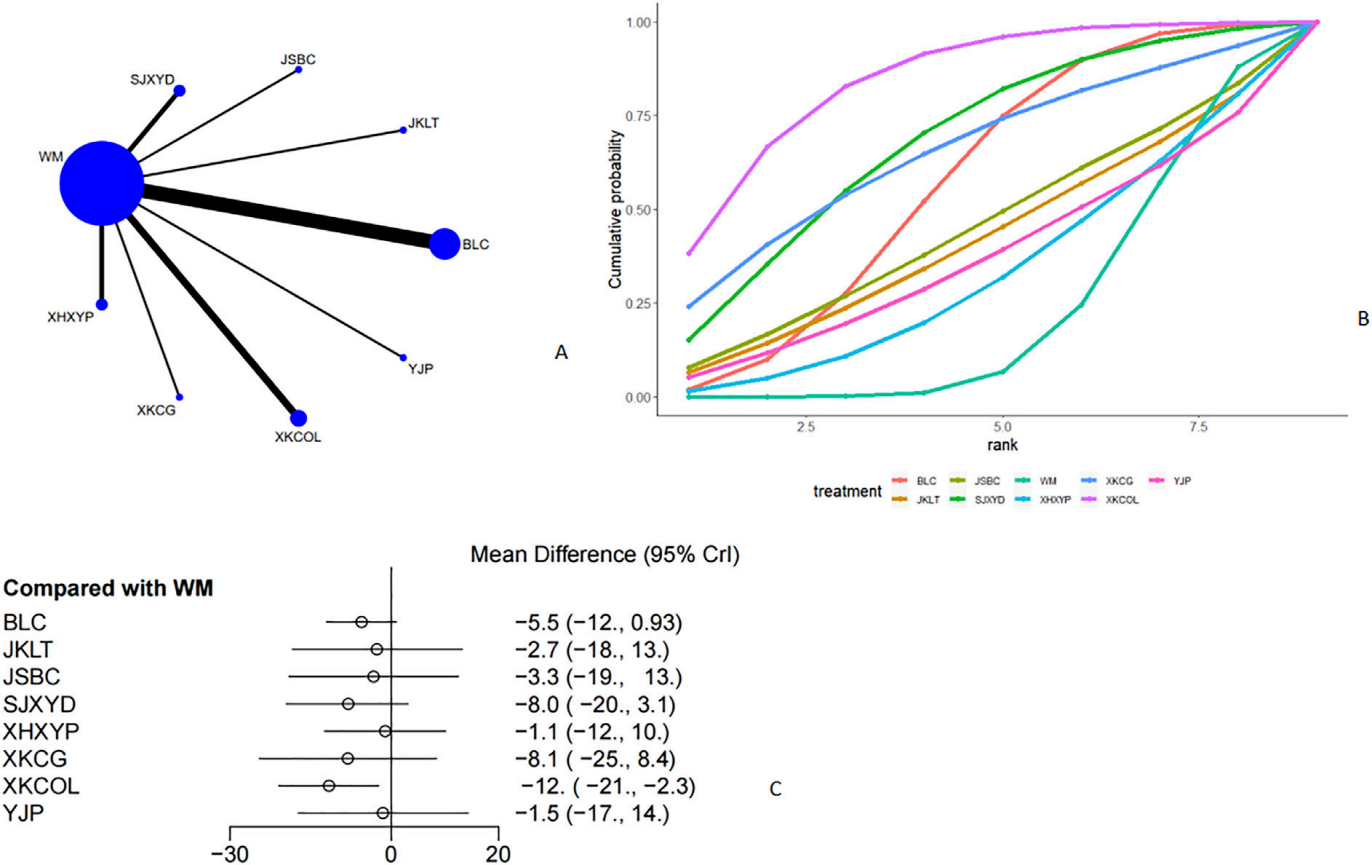
Meta analysis of TRAb (**(A)**: Network plot, **(B)** Area under the cumulative probability curve, **(C)** forest plot).

Seventeen studies (Meng et al., 2019; Haiyan, 2019; Hongjun, 2020; Yidan, 2021; Zhaoli and Xiaobin, 2021; Ming et al., 2014; Hansong et al., 2011a; Minhong and Zhihong, 2016; Zhongyu, 2020; Yaowu, 2017; Min et al., 2019; Lichao et al., 2020; Yi and Xuewei, 2023; Yaowu, 2016; Xiao et al., 2018; Hui et al., 2022; Xinyi et al., 2021) reported TRAb (Figure 10, Figure 10A). From the network plot, it was found that no closed loop was formed, and there was a direct comparison of Western medicine with Sanjie Xiaoying Decoction, Jinshuibao Capsule, Jiakangling Tablet, Bailing Capsule, Yinjia Pellet, *P. vulgaris (L.)* Oral Liquid, Xiehuo Xiaoying Recipe, and *P. vulgaris (L.)* Granules. Compared with Western medicine, *P. vulgaris (L.)* Oral Liquid [MD = −12, 95%CI (−21, −2.3)] could reduce TRAb level (Figure 10C) of GD patients. From the league table, it could be seen that there was no obvious difference among variable Chinese medicines in reducing TRAb level (Supplementary Table S8). According to the SUCRA curve, *P. vulgaris (L.)* Oral Liquid ranked the highest (84.1%), followed by Sanjie Xiaoying Decoction (67.7%), *P. vulgaris (L.)* Granules (65.1%), and Western medicine (22.1%) (Figure 10B; Table 2).

### Heterogeneity test

We tested the heterogeneity for curative effect, TSH, FT3, FT4, TGAb, TPOAb and TRAb. The results showed that in the heterogeneity test of the curative effect, the I^2^ was 17.9% in the pairwise comparison of Western medicine and Bailing Capsule (Supplementary Figures S1, S2 in Supplementary Material S1). In the heterogeneity test of TSH, the I^2^ was 7.9% and 99.1% in the pairwise comparison of Western medicine and Bailing capsule, and of Yikang Pill and Western medicine (Supplementary Figures S3, S4 in Supplementary Material S1). In the heterogeneity test of FT3, the I^2^ was 89.7%, 98.3%, and 97.7%, respectively in the pairwise comparison of Western medicine and Bailing Capsule, of Western medicine and Jiakangling Tablet, and of Yikang Pill and Western medicine (Supplementary Figures S5, S6 in Supplementary Material S1). In the heterogeneity test of FT4, the I^2^ was 94.7%, 62.7%, and 94.0%, respectively in the pairwise comparison of Western medicine and Bailing Capsule, of Western medicine and Jiakangling Tablet, and of Yikang Pill and Western medicine (Supplementary Figures S7, S8 in Supplementary Material S1). In the heterogeneity test of TGAb, the I^2^ was 100% in the pairwise comparison of *P. vulgaris (L.)* Oral Liquid and Western medicine, and of Yikang Pill and Western medicine (Supplementary Figures S9, S10 in Supplementary Material S1). In the heterogeneity test of TPOAb, the I^2^ was 98.4%, 97.6%, and 93.5%, respectively in the pairwise comparison of Western medicine and Bailing Capsule, of *P. vulgaris (L.)* Oral Liquid and Western medicine, and of Yikang Pill and Western medicine (Supplementary Figures S11, S12 in Supplementary Material S1). In the heterogeneity test of TRAb, the I^2^ was 99.9%, 99.8%, 0.0%, and 99.3%, respectively in the pairwise comparison of Western medicine and Bailing Capsule, of Western medicine and Sanjie Xiaoying Decoction, of Xiehuo Xiaoying Recipe and Western medicine, and of *P. vulgaris (L.)* Oral Liquid and Western medicine (Supplementary Figures S13, S14 in Supplementary Material S1).

### Publication bias assessment

We used a funnel plot to evaluate the publication bias of efficacy, TSH, FT3, FT4, TGAb, TPOAb and TRAb, and the results indicated that there was a greater possibility of publication bias in these indicators (Supplementary Figures S15, S21 in Supplementary Material S1).

## Discussion

Graves’ disease, as an autoimmune disease, has extensive influences, and can negatively affect multiple organs. Due to poor nutrition and increased life pressure, the number of patients with this disease is also increasing each year. However, there has been no great progress in the treatment methods, which are mainly drugs, radioactive iodine (^131^I) therapy and surgery. All carry a risk of adverse reactions. Alternatively, traditional Chinese medicine has a significant effect in the adjuvant treatment of GD, and can markedly reduce symptom remission rate and course of treatment, which effectively makes up for the limitations brought by Western medicine. However, there is no systematic research on this aspect at present, so this network meta-analysis compared different traditional Chinese medicines on the curative effect, and levels of TSH, FT3, FT4, TGAb, TPOAb and TRAb in GD patients, which is also the highlight of our study.

This study has found that Yinjia Pellet ranks first in improving curative effect, followed by Common Threewingnut Root. Yikang Pill has the best effect on raising TSH level, followed by *P. vulgaris (L.)* Granules. In improving FT3 level, *P. vulgaris (L.)* Granules has the best effect, followed by Jiakangling Capsule. The effect of *P. vulgaris (L.)* Granules on improving FT4 level is the most significant, followed by Jiakangling Capsule. *Prunella vulgaris (L.)* Oral Liquid has the best effect on reducing TGAb level, followed by Yikang Pill. As to TPOAb level, *P. vulgaris (L.)* Oral Liquid has the best effect, followed by *P. vulgaris (L.)* Granules. In improving the level of TRAb, *P. vulgaris (L.)* Oral Liquid is the first choice, followed by Sanjie Xiaoying Decoction. Modern pharmacological research shows that the main components of Yinjia Pellet, such as Honeysuckle Flower, Rhizoma Coptidis and *P. vulgaris (L.)*, can boost the phagocytosis of white blood cells and inflammatory cells and reduce tumor necrosis factor alpha (TNF-α). Thereby, it can reverse the inflammatory changes of tissues, reduce the toxic effect of natural killer cells, regulate the immune system, reduce the secretion and synthesis of thyroid hormones, and distinctly relieve the symptoms of patients such as anxiety and overeating (Myśliwiec et al., 1999; Jeong et al., 2023; Liu et al., 2022). Tripterygium Wilfordii has anti-inflammatory, anti-allergic and immunosuppressive effects. It can significantly reduce the levels of TNF-α and interleukin-2, inhibit the activation of T cells, regulate cellular immunity and humoral immunity, restore the balance of Th1/Th2 cells and relieve clinical symptoms (Liu et al., 2019). Yikang Pill can reduce the sensitivity of target cells to thyroxine, inhibit lymphocyte infiltration and fibrous tissue proliferation of the thyroid, reduce the damage of thyroid epithelial cells, accelerate the degradation of triiodothyronine *in vivo*, restore the function of the thyroid, and have a benign regulatory effect on the abnormal immune response of thyroid (Shuguang et al., 2010; Kokkinos et al., 2007). In addition, a high dose of thyroid hormone will increase the decomposition of protein and fat, further leading to abnormal secretion of adipocytokines such as adiponectin, serum-free fatty acids and leptin, which will affect glucose and lipid metabolism. Yikang Pill can reduce adiponectin and serum-free fatty acids, regulate leptin secretion, reduce thyroid hormone levels, and improve TSH levels through the feedback mechanism (Pontikides and Krassas, 2007; Biondi, 2010). Both *P. vulgaris (L.)* Granules and *P. vulgaris (L.)* Oral Liquid are *P. vulgaris (L.)* extracts, but their onset time and therapeutic effect are different because of different preparations (Mengqiu et al., 2020). At present, some meta-analyses have proved that *P. vulgaris (L.)* preparations combined with Western medicine can effectively improve thyroid function and reduce autoantibodies (Gao and Li, 2023). *Prunella vulgaris (L.)* polysaccharide is one of the main components of *P. vulgaris (L.)*, which can affect the synthesis and secretion of thyroid hormone. This effect may be achieved by inhibiting the activation of the ERK pathway. *Prunella vulgaris (L.)* polysaccharide can affect the activation of the Raf system, delay the phosphorylation of serine residues in MEK, and thus affect the activation of its substrate ERK1/2, so as to reduce cell proliferation and differentiation (Campbell, 2014; Waseem et al., 2014; Ko et al., 2013). It has been found that *P. vulgaris (L.)* can regulate the function of peripheral lymphocytes, prevent the destruction of follicular cells and further repair damaged follicular cells, so that thyroid function and antibodies can return to normal (Huilin et al., 2020). Some studies have also revealed that *P. vulgaris (L.)* has a significant effect on specific immunity, which can interfere with the Th1/Th2 ratio, downregulate TPOAb, reduce the production of thyroid hormone and regulate the immune state, thus promoting thyroid function (Guo et al., 2021). Jiakangling Capsule can target the transduction pathway of Akt signaling, regulate cell proliferation and apoptosis, inhibit the over-phosphorylation of mTOP to some extent, affect the transcription activation of proliferation-associated genes by downstream signaling pathways, improve thyroid function and regulate immunity (Fingar and Blenis, 2004; Zhang et al., 2023; Li et al., 2015). Other studies have shown that Jiakangling Capsule can coordinate with HPT axis, play a positive role in regulating TSH, and reduce the synthesis and secretion of thyroid hormone through feedback mechanism, so as to improve the thyroid function of patients (Yan, 2022). Sanjie Xiaoying Decoction has an obvious effect on reducing TRAb, which can alleviate the stimulation to thyroid cells and downregulate HPT axis through feedback, improve thyroid function and reduce the recurrence rate (Zhongyu, 2020).

## Limitations

In this study, we discussed the disparities of different traditional Chinese medicines in the adjuvant treatment of Graves’ disease, but we found that there were no significant differences among these top intervention measures in the league table. Hence, more research is warranted to validate our view on the option of traditional Chinese medicines.

There are still some limitations in this study. Firstly, the literature quality is low, and most of the included studies are not registered, of which the task concealment and blinding method were not described. This limits the reliability of the results. Secondly, the included original research is subjective in the evaluation of clinical efficacy, and there may be publication bias and implementation bias. Thirdly, the included studies are all in Chinese, which reduces the extrapolation capability of the results. Fourthly, different dosage forms of traditional Chinese medicine are not uniform, so there may be errors in the research conclusions. Fifthly, most of the studies fail to include the follow-up to patients and pay little attention to the long-term efficacy of drugs. Sixth, the sample sizes of studies on different traditional Chinese medicines included in this analysis are different. Therefore, in order to draw more accurate conclusions, more high-quality randomized controlled trials are needed.

## Conclusion

Based on the current research, it is safe to conclude that traditional Chinese medicine can improve the curative effect and TSH level of patients with Graves’ disease, and reduce the levels of FT3, FT4, TPOAb and TRAb. Besides, Yinjia Pellet is the most helpful in improving the curative effect. Yikang Pill can best improve TSH. *Prunella vulgaris (L.)* Granules have the best effect on reducing FT3. *Prunella vulgaris (L.)* Granules perform best in reducing FT4. *Prunella vulgaris (L.)* Oral Liquid has the most favorable effect on reducing TPOAb and TRAb. Therefore, in future clinical and scientific research practices, Yinjia Pellet, *P. vulgaris (L.)* Granules, and *P. vulgaris (L.)* Oral Liquid can be further investigated, thus promoting the development of new drugs.

## Data Availability

The original contributions presented in the study are included in the article/Supplementary Material, further inquiries can be directed to the corresponding author.

## Supplementary material

SUPPLEMENTARY FIGURE S1 Heterogeneity test 1 of curative effect.

SUPPLEMENTARY FIGURE S2 Heterogeneity test 2 of curative effect.

SUPPLEMENTARY FIGURE S3 Heterogeneity test 1 of TSH.

SUPPLEMENTARY FIGURE S4 Heterogeneity test 2 of TSH.

SUPPLEMENTARY FIGURE S5 Heterogeneity test 1 of FT3.

SUPPLEMENTARY FIGURE S6 Heterogeneity test 2 of FT3.

SUPPLEMENTARY FIGURE S7 Heterogeneity test 1 of FT4.

SUPPLEMENTARY FIGURE S8 Heterogeneity test 2 of FT4.

SUPPLEMENTARY FIGURE S9 Heterogeneity test 1 of TGAb.

SUPPLEMENTARY FIGURE S10 Heterogeneity test 2 of TGAb.

SUPPLEMENTARY FIGURE S11 Heterogeneity test 1 of TPOAb.

SUPPLEMENTARY FIGURE S12 Heterogeneity test 2 of TPOAb.

SUPPLEMENTARY FIGURE S13 Heterogeneity test 1 of TRAb.

SUPPLEMENTARY FIGURE S14 Heterogeneity test 2 of TRAb.

SUPPLEMENTARY FIGURE S15 Curative effect funnel plot.

SUPPLEMENTARY FIGURE S16 TSH funnel plot.

SUPPLEMENTARY FIGURE S17 FT3 funnel plot.

SUPPLEMENTARY FIGURE S18 FT4 funnel plot.

SUPPLEMENTARY FIGURE S19 TGAb funnel plot.

SUPPLEMENTARY FIGURE S20 TPOAb funnel plot.

SUPPLEMENTARY FIGURE S21 TRAb funnel plot.

SUPPLEMENTARY TABLE S1 Search strategy.

SUPPLEMENTARY TABLE S2 Curative effect league table.

SUPPLEMENTARY TABLE S3 TSH league table.

SUPPLEMENTARY TABLE S4 FT3 league table.

SUPPLEMENTARY TABLE S5 FT4 league table.

SUPPLEMENTARY TABLE S6 TGAb league table.

SUPPLEMENTARY TABLE S7 TPOAb league table.

SUPPLEMENTARY TABLE S8 TRAb league table.

SUPPLEMENTARY MATERIAL S3 Preparations of Chinese medicine.
