# Supplementary material for: Effect of traditional Chinese medicine on Graves’ disease: a network meta-analysis
Source: Front Pharmacol. 2024 Aug 22;15:1411459. doi: 10.3389/fphar.2024.1411459 (PMC11374712; doi:10.3389/fphar.2024.1411459)
Supplement: Supplementary file 2 [file Table1.DOCX]

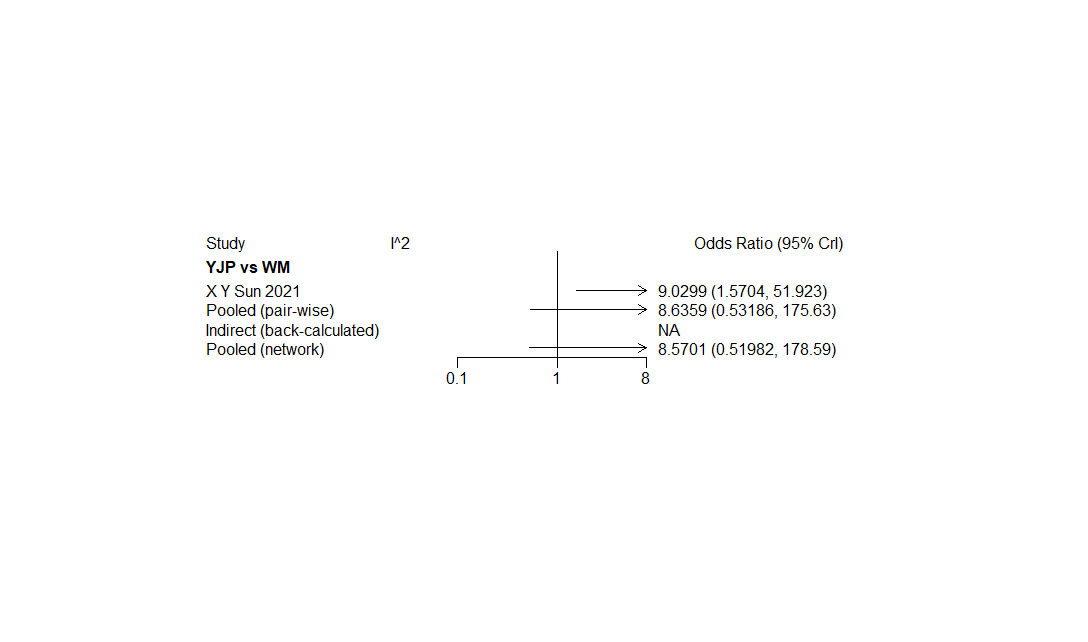

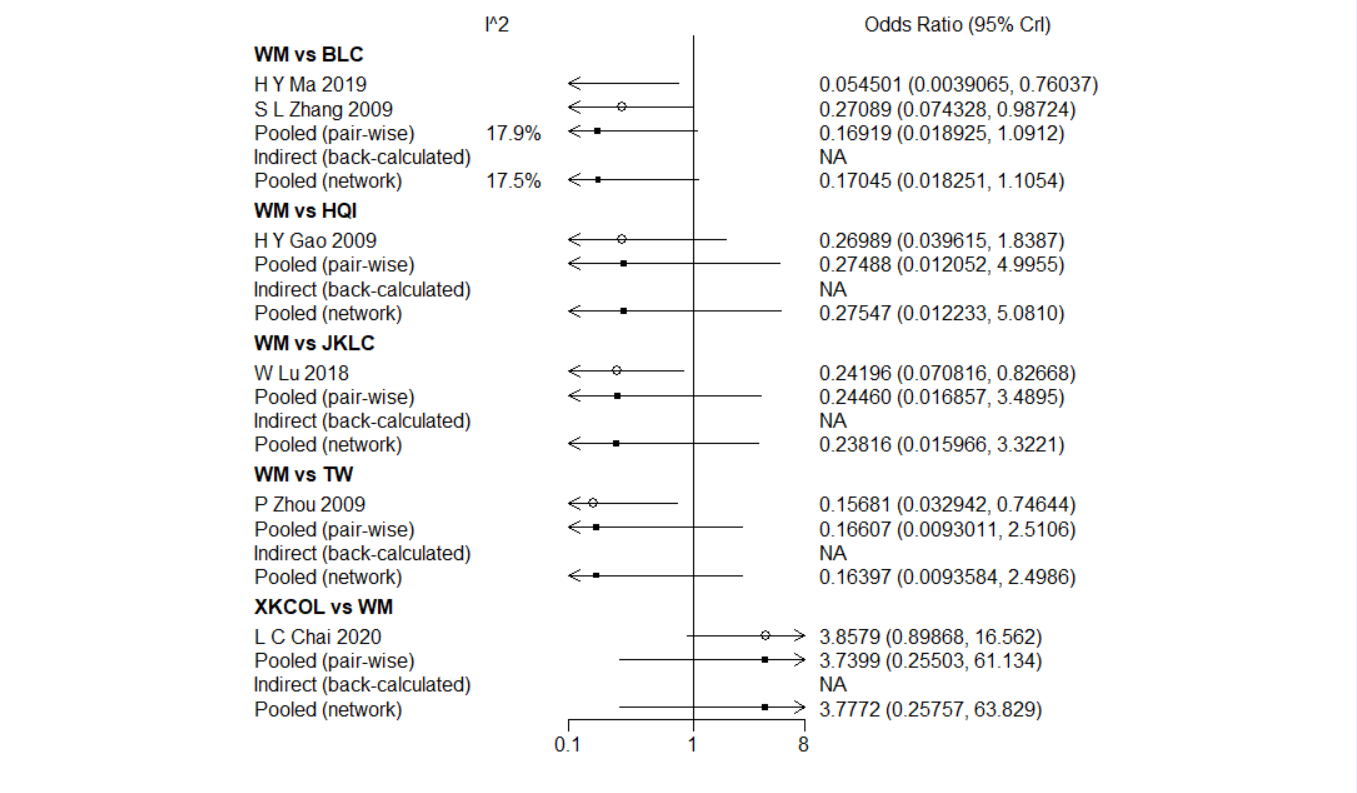
Figure S1: Heterogeneity test (1) for efficacy.

Figure S2: Heterogeneity test (2) for efficacy.


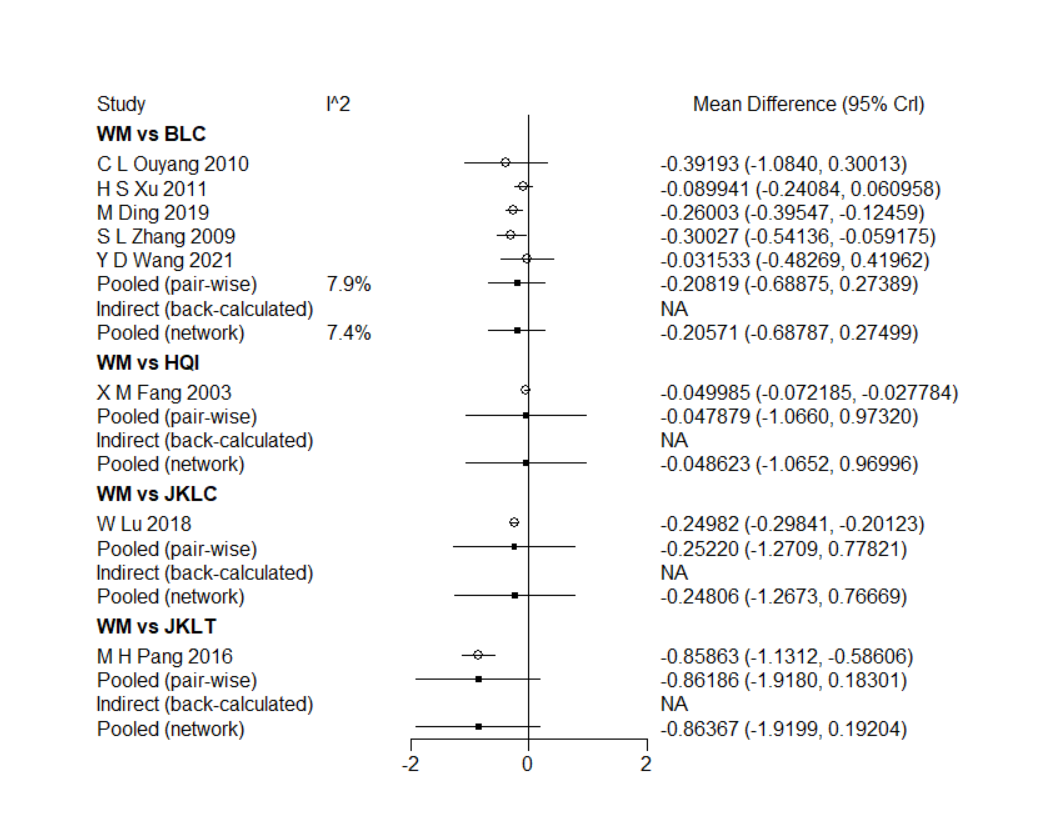


Figure S3: Heterogeneity test (1) for TSH.


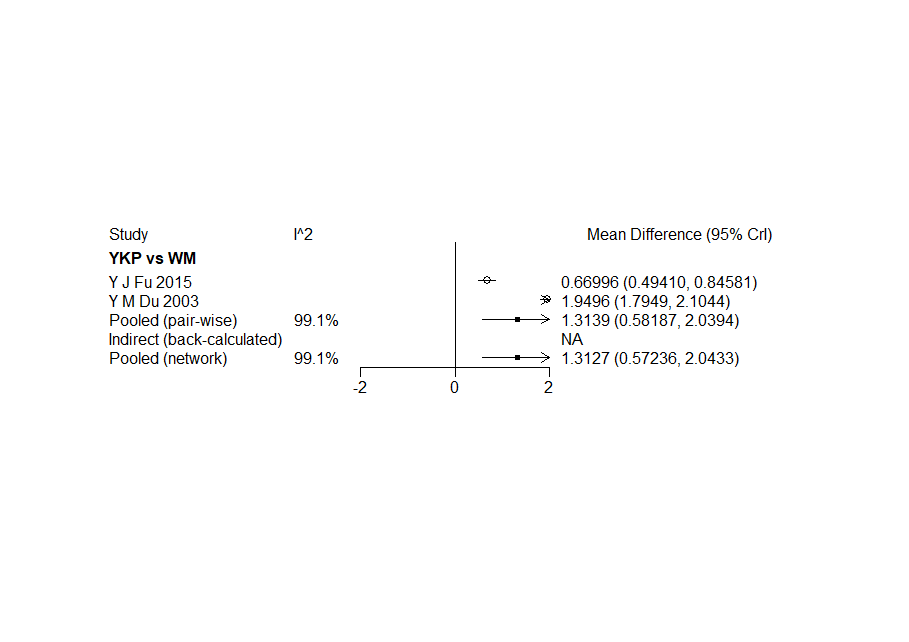


Figure S4: Heterogeneity test (2) for TSH.


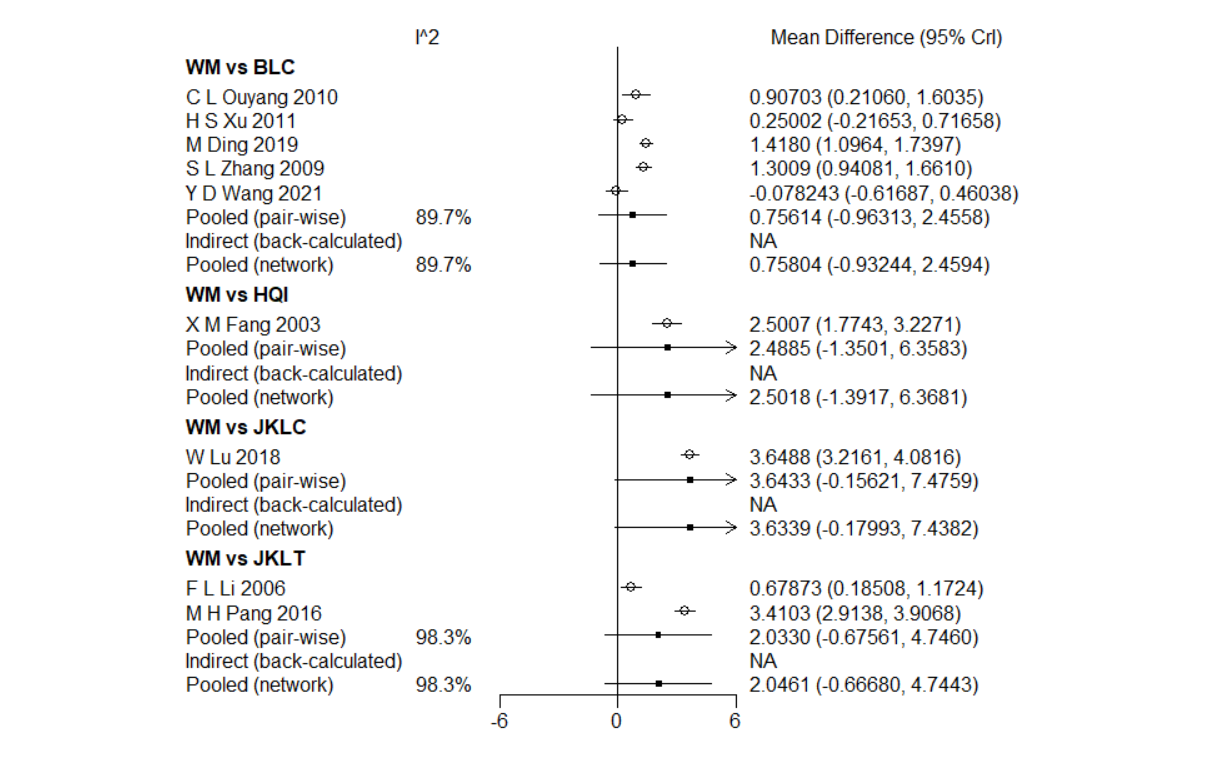


Figure S5: Heterogeneity test (1) for FT3.


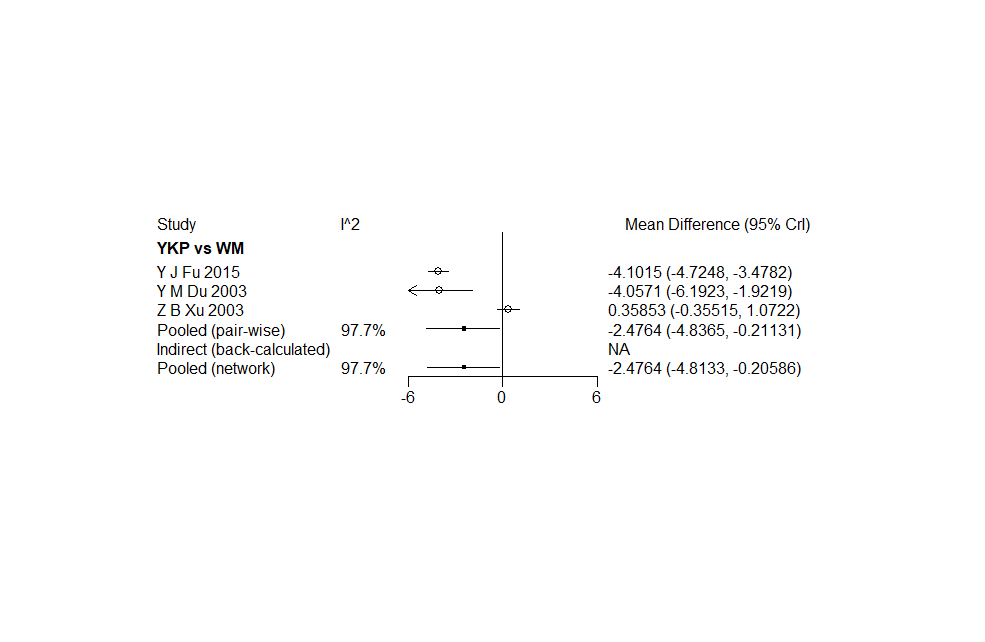


Figure S6: Heterogeneity test (2) for FT3.


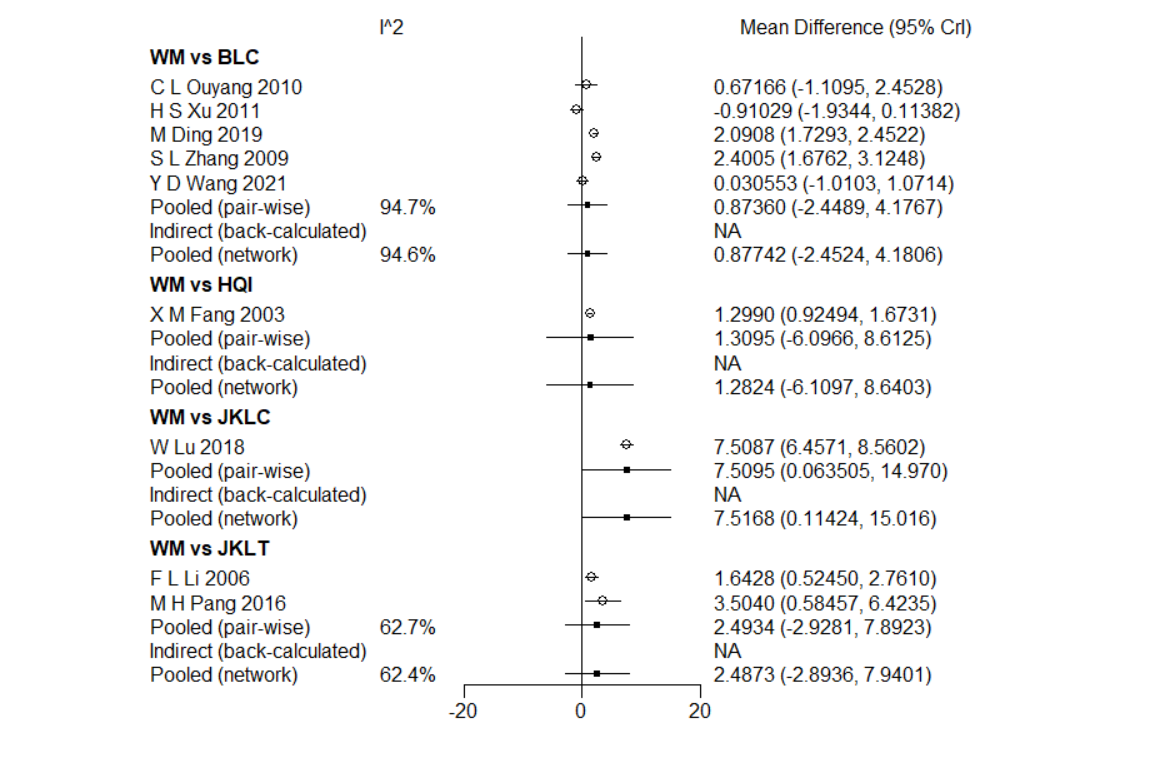


Figure
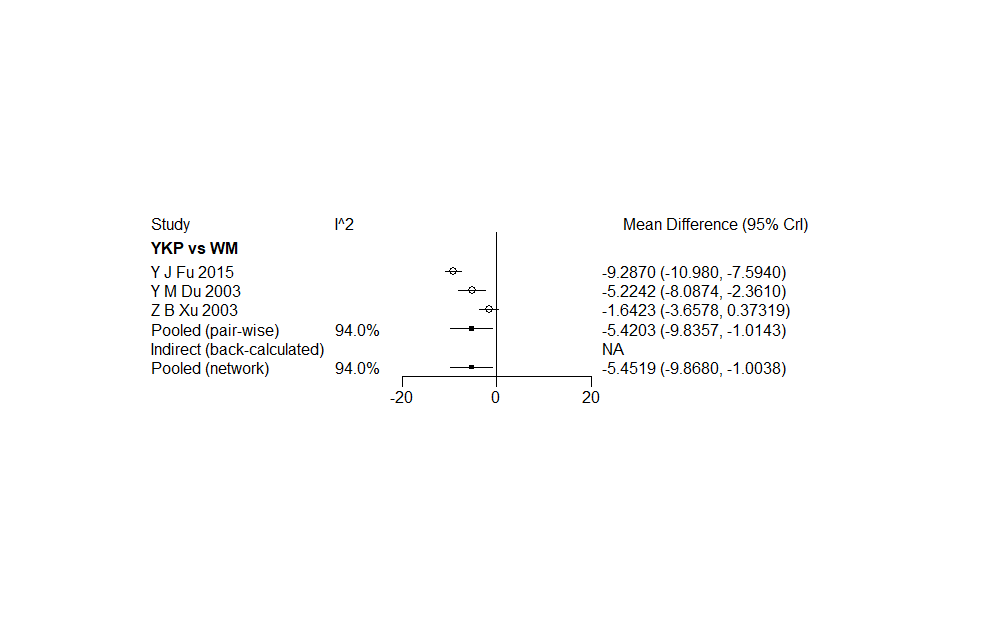
S7: Heterogeneity test (1) for FT4.


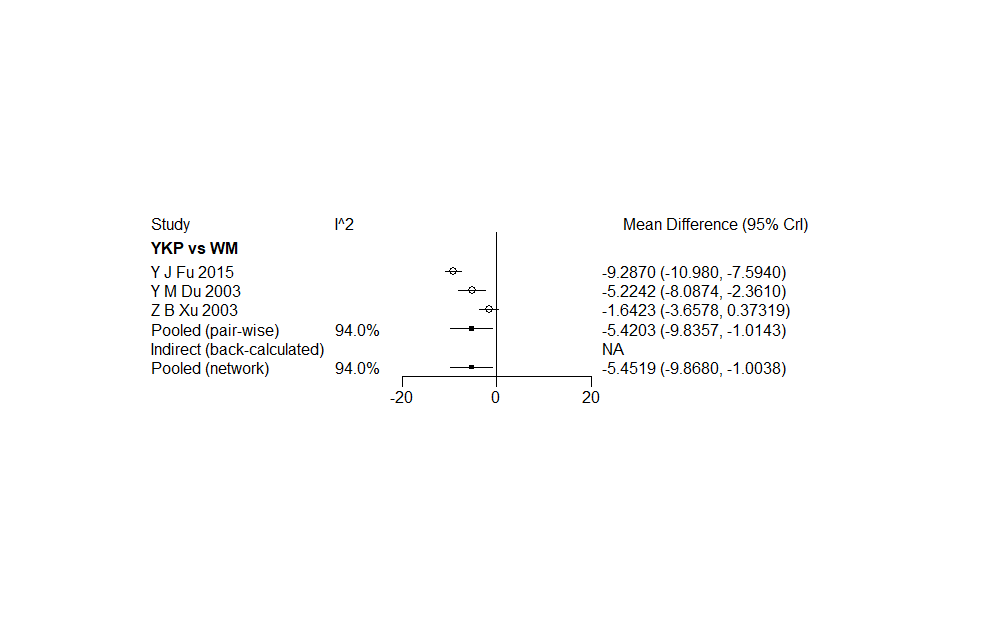


Figure S8: Heterogeneity test (2) for FT4.


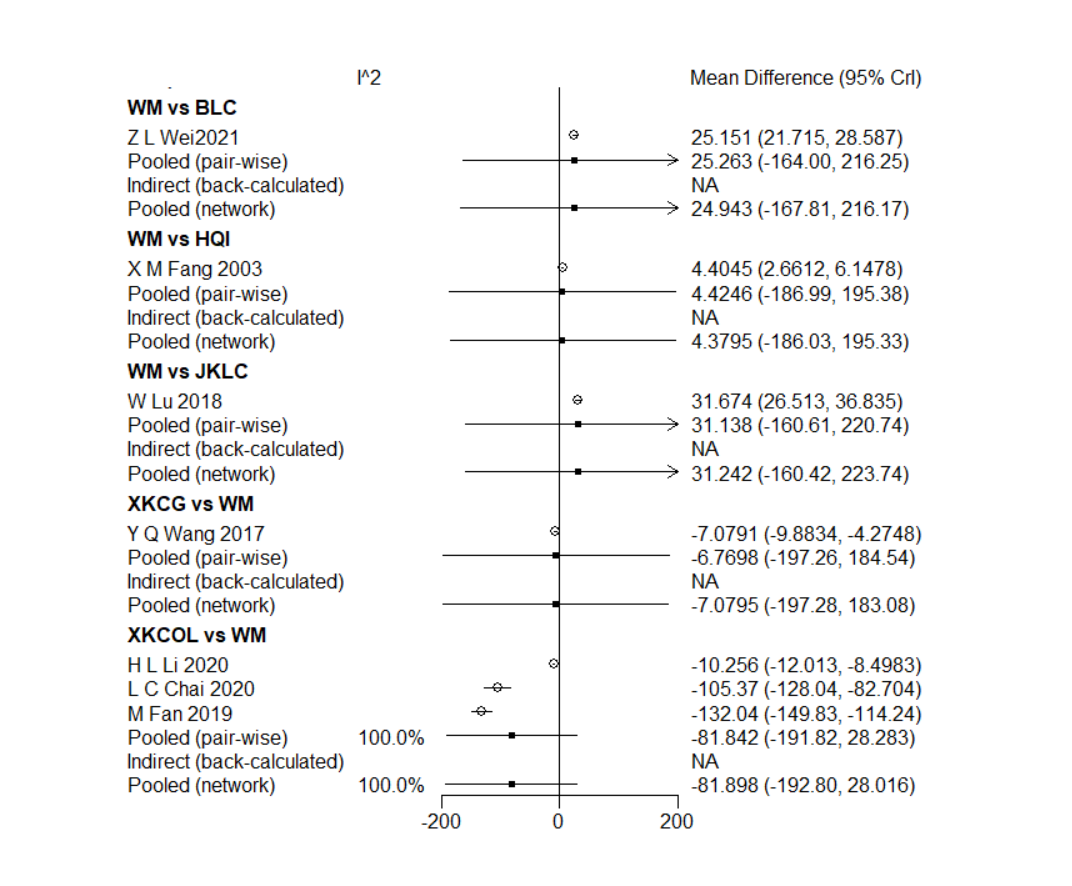


Figure S9: Heterogeneity test (1) for TGAb.


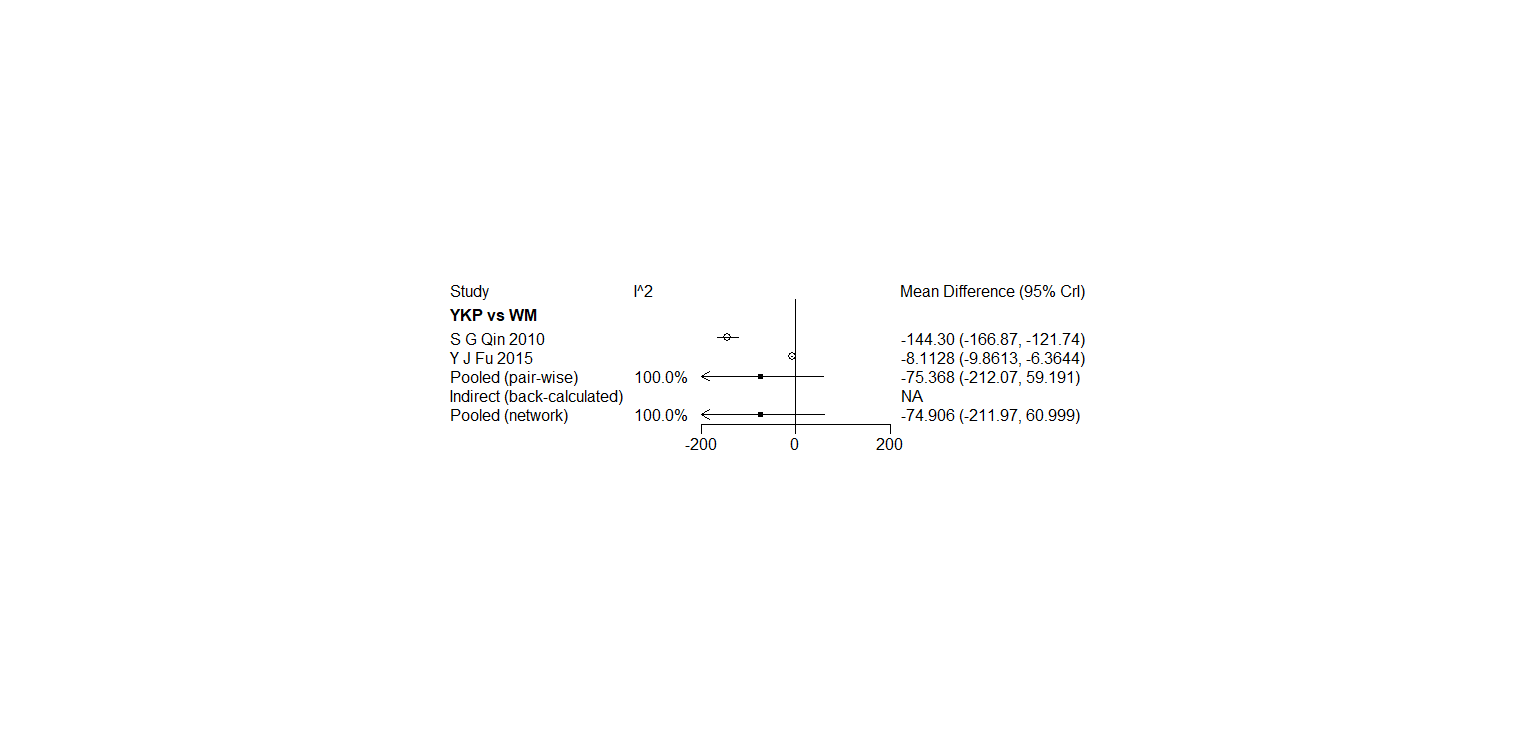


Figure S10: Heterogeneity test (2) for TGAb.


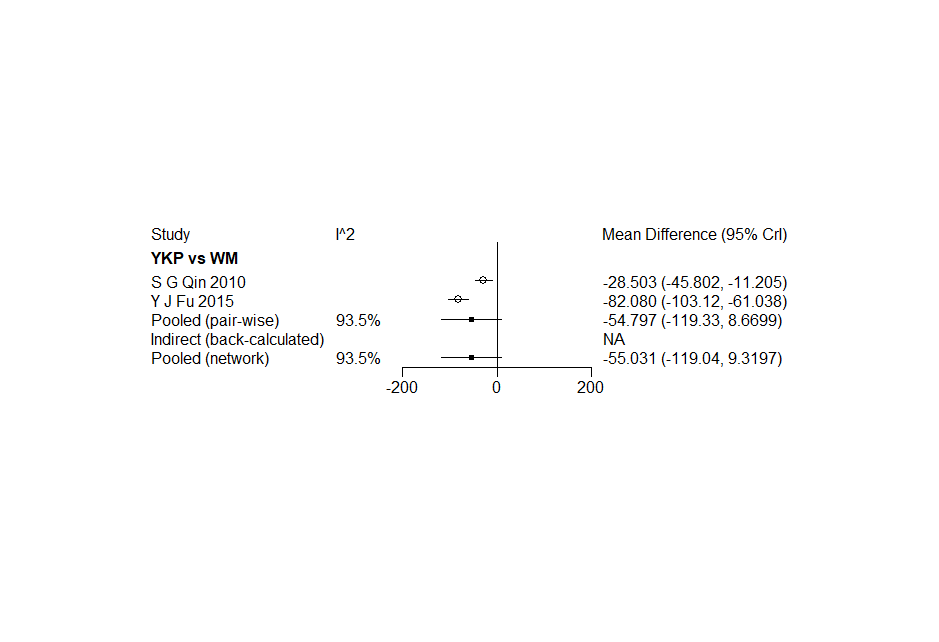

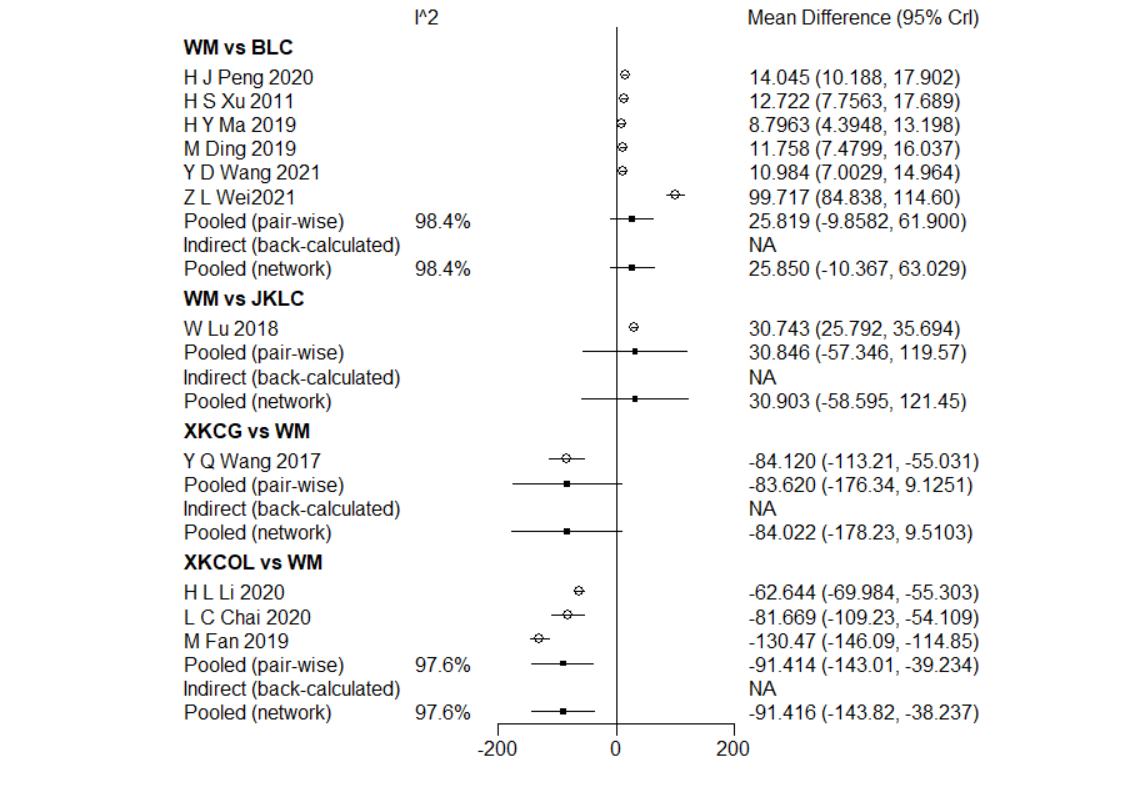


Figure S11: Heterogeneity test (1) for TPOAb.

Figure S12: Heterogeneity test (2) for TPOAb.


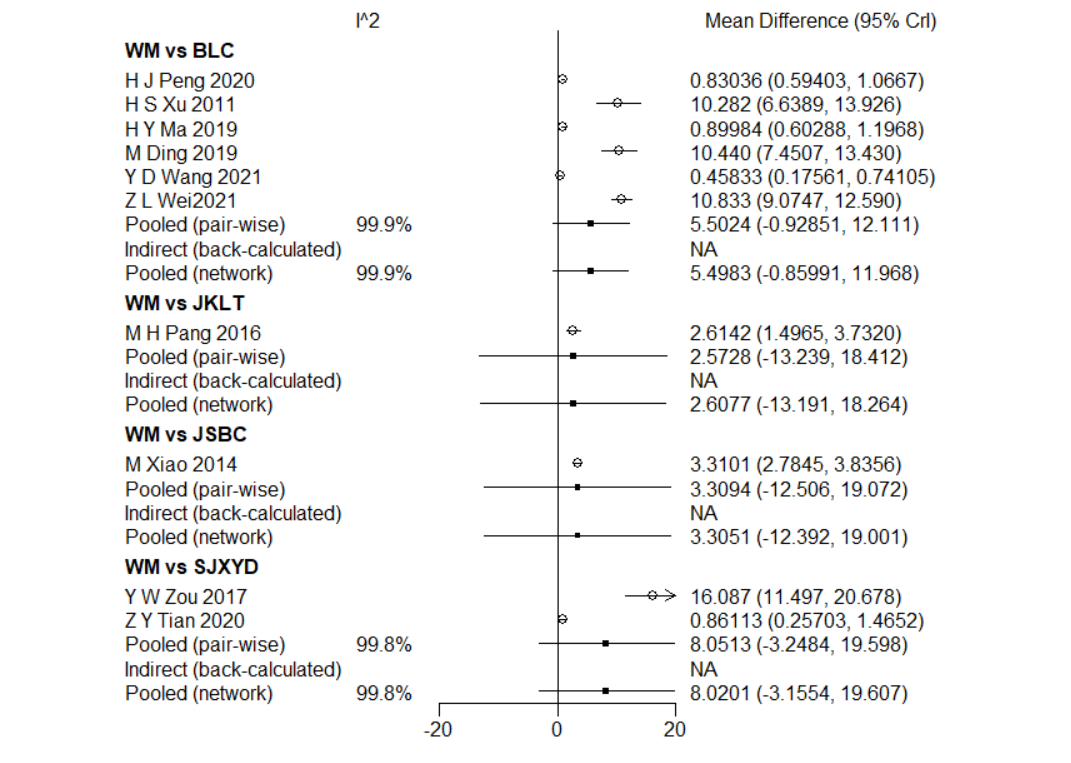


Figure S13: Heterogeneity test (1) for TRAb.


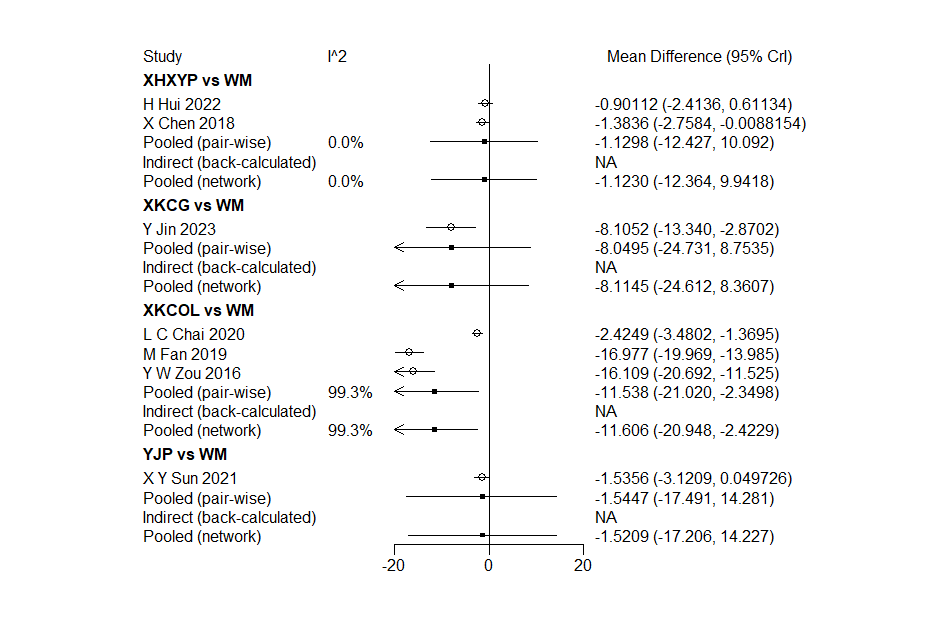


Figure S14: Heterogeneity test (2) for TRAb.


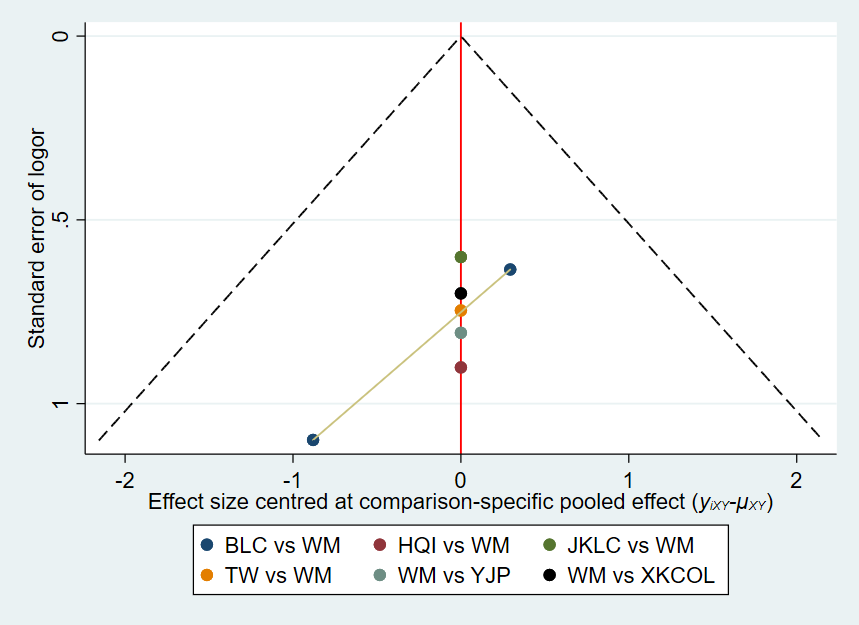


Figure S15: Funnel plot for efficacy.


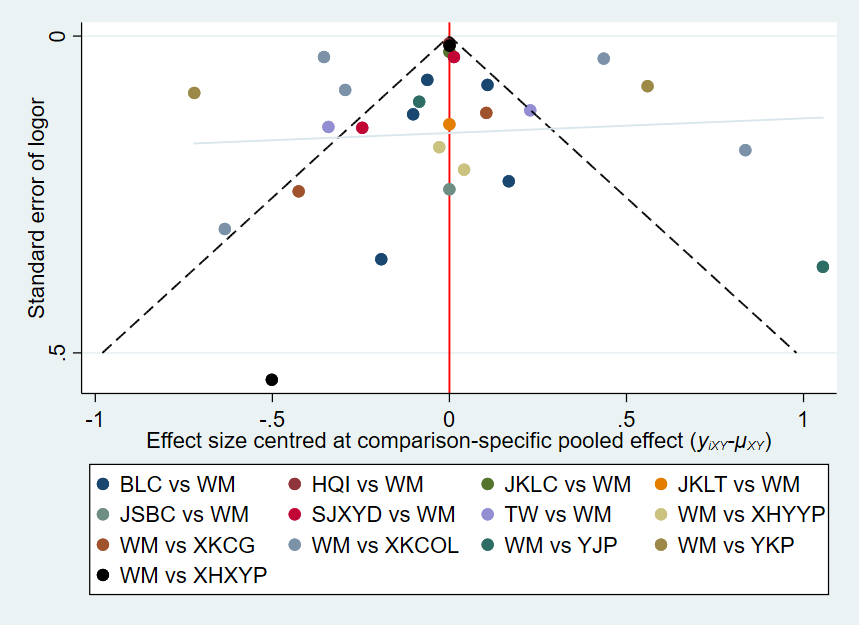


Figure S16: Funnel plot for TSH.


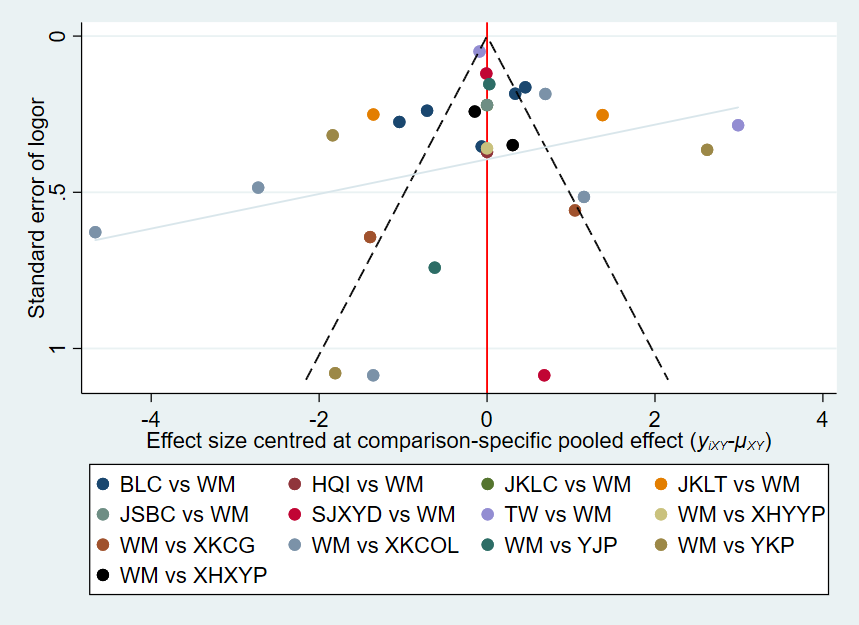


Figure S17: Funnel plot for FT3.


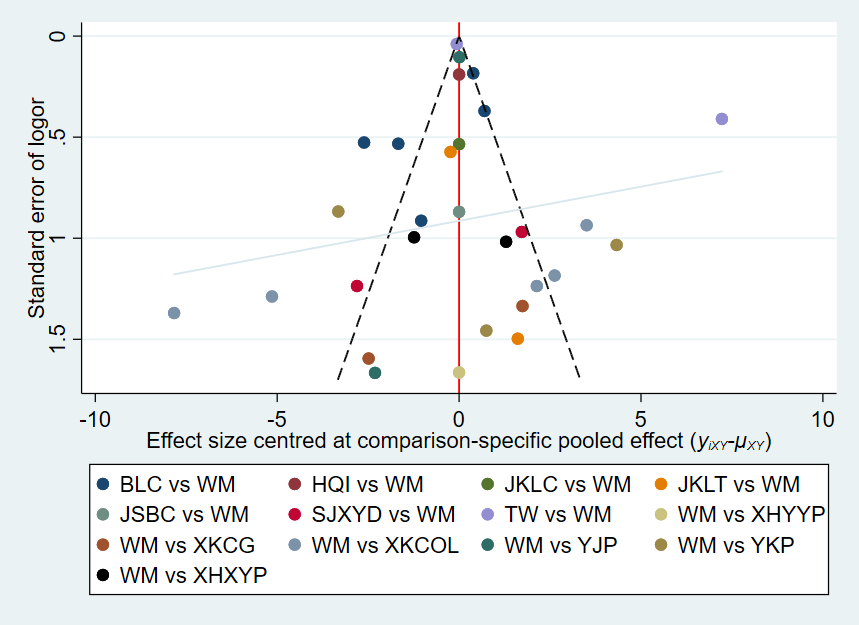


Figure S18: Funnel plot for FT4.


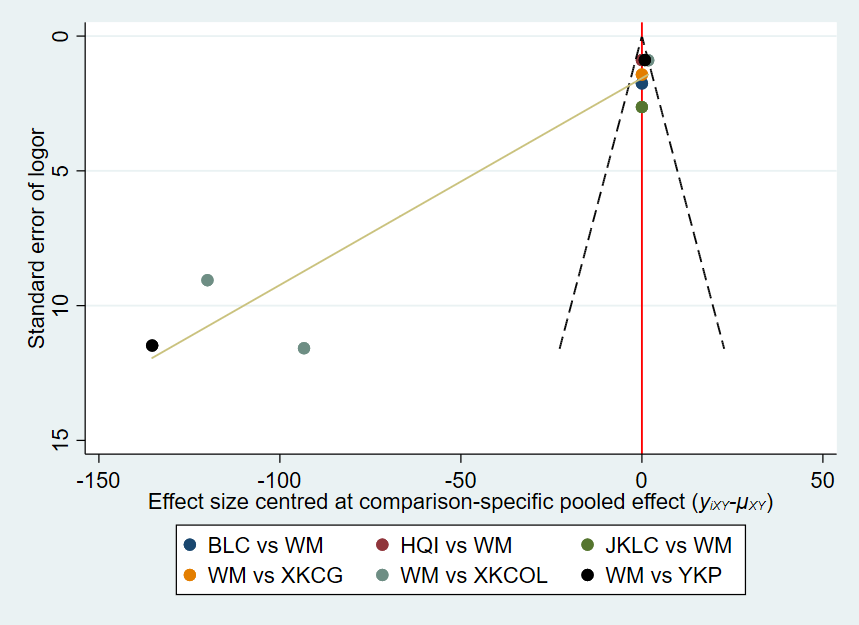


Figure S19: Funnel plot for TGAb.


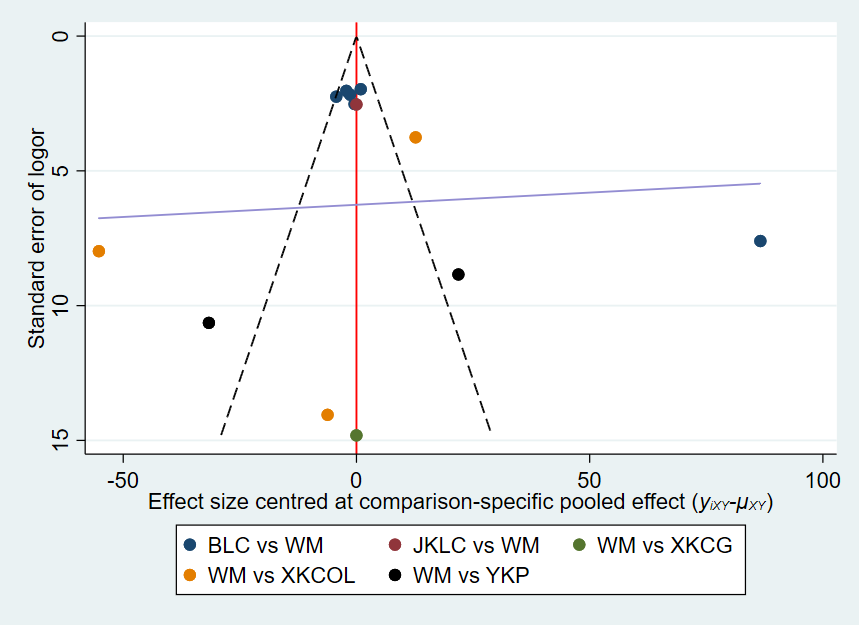


Figure S20: Funnel plot for TPOAb.


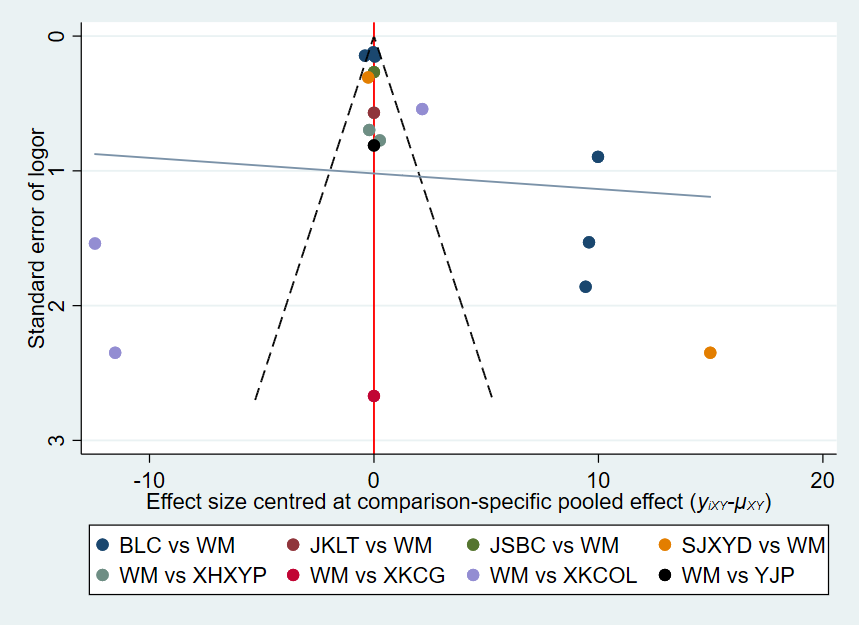


Figure S21: Funnel plot for TRAb.
