# Supplementary material for: Effect of traditional Chinese medicine on Graves’ disease: a network meta-analysis
Source: Front Pharmacol. 2024 Aug 22;15:1411459. doi: 10.3389/fphar.2024.1411459 (PMC11374712; doi:10.3389/fphar.2024.1411459)
Supplement: Supplementary file 3 [file Table2.DOCX]

**Table S1:** Search strategy.

**Pubmed:**

Search number Query Sort By Filters Search Details Results Time

7 (("Graves Disease"[Mesh]) OR ((((((((((((((Graves Disease[Title/Abstract]) OR (Disease, Graves[Title/Abstract])) OR (Basedow Disease[Title/Abstract])) OR (Disease, Basedow[Title/Abstract])) OR (Graves' Disease[Title/Abstract])) OR (Disease, Graves'[Title/Abstract])) OR (Exophthalmic Goiter[Title/Abstract])) OR (Exophthalmic Goiters[Title/Abstract])) OR (Goiters, Exophthalmic[Title/Abstract])) OR (Goiter, Exophthalmic[Title/Abstract])) OR (Hyperthyroidism, Autoimmune[Title/Abstract])) OR (Basedow's Disease[Title/Abstract])) OR (Basedows Disease[Title/Abstract])) OR (Disease, Basedow's[Title/Abstract]))) AND (("Medicine, Chinese Traditional"[Mesh]) OR (((((((((((((((((((((((Medicine, Chinese Traditional[Title/Abstract]) OR (Traditional Chinese Medicine[Title/Abstract])) OR (Chung I Hsueh[Title/Abstract])) OR (Hsueh, Chung I[Title/Abstract])) OR (Traditional Medicine, Chinese[Title/Abstract])) OR (Zhong Yi XueZhong Yi Xue[Title/Abstract])) OR (Chinese Traditional Medicine[Title/Abstract])) OR (Chinese Medicine, Traditional[Title/Abstract])) OR (Traditional Tongue Diagnosis[Title/Abstract])) OR (Tongue Diagnoses, Traditional[Title/Abstract])) OR (Tongue Diagnosis, Traditional[Title/Abstract])) OR (Traditional Tongue Diagnoses[Title/Abstract])) OR (Traditional Tongue Assessment[Title/Abstract])) OR (Tongue Assessment, Traditional[Title/Abstract])) OR (Traditional Tongue Assessments[Title/Abstract])) OR (Tang[Title/Abstract])) OR (Decoction[Title/Abstract])) OR (Fang[Title/Abstract])) OR (Wan[Title/Abstract])) OR (Keli[Title/Abstract])) OR (San[Title/Abstract])) OR (Zhusheye[Title/Abstract])) OR (Injection[Title/Abstract]))) ("Graves Disease"[MeSH Terms] OR ("Graves Disease"[Title/Abstract] OR "disease graves"[Title/Abstract] OR "basedow disease"[Title/Abstract] OR "disease basedow"[Title/Abstract] OR "Graves Disease"[Title/Abstract] OR "disease graves"[Title/Abstract] OR "exophthalmic goiter"[Title/Abstract] OR "exophthalmic goiters"[Title/Abstract] OR (("Goiter"[MeSH Terms] OR "Goiter"[All Fields] OR "Goiters"[All Fields] OR "goitres"[All Fields] OR "goiterous"[All Fields] OR "goitre"[All Fields]) AND "Exophthalmic"[Title/Abstract]) OR "goiter exophthalmic"[Title/Abstract] OR "hyperthyroidism autoimmune"[Title/Abstract] OR "basedow s disease"[Title/Abstract] OR "basedows disease"[Title/Abstract] OR "disease basedow s"[Title/Abstract])) AND ("medicine, chinese traditional"[MeSH Terms] OR ("medicine chinese traditional"[Title/Abstract] OR "traditional chinese medicine"[Title/Abstract] OR ((chung i[Author] OR chung i[Investigator]) AND "Hsueh"[Title/Abstract]) OR ("Hsueh"[All Fields] AND "chung i"[Title/Abstract]) OR "traditional medicine chinese"[Title/Abstract] OR ((((zhong, yi[Author] OR yi, zhong[Author]) OR zhong yi[Author] OR zhong yi[Investigator]) AND "XueZhong"[All Fields]) AND "yi xue"[Title/Abstract]) OR "chinese traditional medicine"[Title/Abstract] OR "chinese medicine traditional"[Title/Abstract] OR "traditional tongue diagnosis"[Title/Abstract] OR ((("Tongue"[MeSH Terms] OR "Tongue"[All Fields] OR "tongues"[All Fields] OR "tongue s"[All Fields]) AND ("diagnosable"[All Fields] OR "diagnosi"[All Fields] OR "Diagnosis"[MeSH Terms] OR "Diagnosis"[All Fields] OR "diagnose"[All Fields] OR "diagnosed"[All Fields] OR "Diagnoses"[All Fields] OR "diagnosing"[All Fields] OR "Diagnosis"[MeSH Subheading])) AND "Traditional"[Title/Abstract]) OR ((("Tongue"[MeSH Terms] OR "Tongue"[All Fields] OR "tongues"[All Fields] OR "tongue s"[All Fields]) AND ("diagnosable"[All Fields] OR "diagnosi"[All Fields] OR "Diagnosis"[MeSH Terms] OR "Diagnosis"[All Fields] OR "diagnose"[All Fields] OR "diagnosed"[All Fields] OR "Diagnoses"[All Fields] OR "diagnosing"[All Fields] OR "Diagnosis"[MeSH Subheading])) AND "Traditional"[Title/Abstract]) OR (("tradition"[All Fields] OR "tradition s"[All Fields] OR "Traditional"[All Fields] OR "traditionals"[All Fields] OR "traditions"[All Fields]) AND "tongue diagnoses"[Title/Abstract]) OR (("tradition"[All Fields] OR "tradition s"[All Fields] OR "Traditional"[All Fields] OR "traditionals"[All Fields] OR "traditions"[All Fields]) AND "tongue assessment"[Title/Abstract]) OR (("Tongue"[MeSH Terms] OR "Tongue"[All Fields] OR "tongues"[All Fields] OR "tongue s"[All Fields]) AND "assessment traditional"[Title/Abstract]) OR (("tradition"[All Fields] OR "tradition s"[All Fields] OR "Traditional"[All Fields] OR "traditionals"[All Fields] OR "traditions"[All Fields]) AND "tongue assessments"[Title/Abstract]) OR "Tang"[Title/Abstract] OR "Decoction"[Title/Abstract] OR "Fang"[Title/Abstract] OR "Wan"[Title/Abstract] OR "Keli"[Title/Abstract] OR "San"[Title/Abstract] OR "Zhusheye"[Title/Abstract] OR "Injection"[Title/Abstract])) 308 20:04:52

6 ("Medicine, Chinese Traditional"[Mesh]) OR (((((((((((((((((((((((Medicine, Chinese Traditional[Title/Abstract]) OR (Traditional Chinese Medicine[Title/Abstract])) OR (Chung I Hsueh[Title/Abstract])) OR (Hsueh, Chung I[Title/Abstract])) OR (Traditional Medicine, Chinese[Title/Abstract])) OR (Zhong Yi XueZhong Yi Xue[Title/Abstract])) OR (Chinese Traditional Medicine[Title/Abstract])) OR (Chinese Medicine, Traditional[Title/Abstract])) OR (Traditional Tongue Diagnosis[Title/Abstract])) OR (Tongue Diagnoses, Traditional[Title/Abstract])) OR (Tongue Diagnosis, Traditional[Title/Abstract])) OR (Traditional Tongue Diagnoses[Title/Abstract])) OR (Traditional Tongue Assessment[Title/Abstract])) OR (Tongue Assessment, Traditional[Title/Abstract])) OR (Traditional Tongue Assessments[Title/Abstract])) OR (Tang[Title/Abstract])) OR (Decoction[Title/Abstract])) OR (Fang[Title/Abstract])) OR (Wan[Title/Abstract])) OR (Keli[Title/Abstract])) OR (San[Title/Abstract])) OR (Zhusheye[Title/Abstract])) OR (Injection[Title/Abstract])) "medicine, chinese traditional"[MeSH Terms] OR ("medicine chinese traditional"[Title/Abstract] OR "traditional chinese medicine"[Title/Abstract] OR ((chung i[Author] OR chung i[Investigator]) AND "Hsueh"[Title/Abstract]) OR ("Hsueh"[All Fields] AND "chung i"[Title/Abstract]) OR "traditional medicine chinese"[Title/Abstract] OR ((((zhong, yi[Author] OR yi, zhong[Author]) OR zhong yi[Author] OR zhong yi[Investigator]) AND "XueZhong"[All Fields]) AND "yi xue"[Title/Abstract]) OR "chinese traditional medicine"[Title/Abstract] OR "chinese medicine traditional"[Title/Abstract] OR "traditional tongue diagnosis"[Title/Abstract] OR ((("Tongue"[MeSH Terms] OR "Tongue"[All Fields] OR "tongues"[All Fields] OR "tongue s"[All Fields]) AND ("diagnosable"[All Fields] OR "diagnosi"[All Fields] OR "Diagnosis"[MeSH Terms] OR "Diagnosis"[All Fields] OR "diagnose"[All Fields] OR "diagnosed"[All Fields] OR "Diagnoses"[All Fields] OR "diagnosing"[All Fields] OR "Diagnosis"[MeSH Subheading])) AND "Traditional"[Title/Abstract]) OR ((("Tongue"[MeSH Terms] OR "Tongue"[All Fields] OR "tongues"[All Fields] OR "tongue s"[All Fields]) AND ("diagnosable"[All Fields] OR "diagnosi"[All Fields] OR "Diagnosis"[MeSH Terms] OR "Diagnosis"[All Fields] OR "diagnose"[All Fields] OR "diagnosed"[All Fields] OR "Diagnoses"[All Fields] OR "diagnosing"[All Fields] OR "Diagnosis"[MeSH Subheading])) AND "Traditional"[Title/Abstract]) OR (("tradition"[All Fields] OR "tradition s"[All Fields] OR "Traditional"[All Fields] OR "traditionals"[All Fields] OR "traditions"[All Fields]) AND "tongue diagnoses"[Title/Abstract]) OR (("tradition"[All Fields] OR "tradition s"[All Fields] OR "Traditional"[All Fields] OR "traditionals"[All Fields] OR "traditions"[All Fields]) AND "tongue assessment"[Title/Abstract]) OR (("Tongue"[MeSH Terms] OR "Tongue"[All Fields] OR "tongues"[All Fields] OR "tongue s"[All Fields]) AND "assessment traditional"[Title/Abstract]) OR (("tradition"[All Fields] OR "tradition s"[All Fields] OR "Traditional"[All Fields] OR "traditionals"[All Fields] OR "traditions"[All Fields]) AND "tongue assessments"[Title/Abstract]) OR "Tang"[Title/Abstract] OR "Decoction"[Title/Abstract] OR "Fang"[Title/Abstract] OR "Wan"[Title/Abstract] OR "Keli"[Title/Abstract] OR "San"[Title/Abstract] OR "Zhusheye"[Title/Abstract] OR "Injection"[Title/Abstract]) 645,597 20:02:31

5 ((((((((((((((((((((((Medicine, Chinese Traditional[Title/Abstract]) OR (Traditional Chinese Medicine[Title/Abstract])) OR (Chung I Hsueh[Title/Abstract])) OR (Hsueh, Chung I[Title/Abstract])) OR (Traditional Medicine, Chinese[Title/Abstract])) OR (Zhong Yi XueZhong Yi Xue[Title/Abstract])) OR (Chinese Traditional Medicine[Title/Abstract])) OR (Chinese Medicine, Traditional[Title/Abstract])) OR (Traditional Tongue Diagnosis[Title/Abstract])) OR (Tongue Diagnoses, Traditional[Title/Abstract])) OR (Tongue Diagnosis, Traditional[Title/Abstract])) OR (Traditional Tongue Diagnoses[Title/Abstract])) OR (Traditional Tongue Assessment[Title/Abstract])) OR (Tongue Assessment, Traditional[Title/Abstract])) OR (Traditional Tongue Assessments[Title/Abstract])) OR (Tang[Title/Abstract])) OR (Decoction[Title/Abstract])) OR (Fang[Title/Abstract])) OR (Wan[Title/Abstract])) OR (Keli[Title/Abstract])) OR (San[Title/Abstract])) OR (Zhusheye[Title/Abstract])) OR (Injection[Title/Abstract]) "medicine chinese traditional"[Title/Abstract] OR "traditional chinese medicine"[Title/Abstract] OR ((chung i[Author] OR chung i[Investigator]) AND "Hsueh"[Title/Abstract]) OR ("Hsueh"[All Fields] AND "chung i"[Title/Abstract]) OR "traditional medicine chinese"[Title/Abstract] OR ((((zhong, yi[Author] OR yi, zhong[Author]) OR zhong yi[Author] OR zhong yi[Investigator]) AND "XueZhong"[All Fields]) AND "yi xue"[Title/Abstract]) OR "chinese traditional medicine"[Title/Abstract] OR "chinese medicine traditional"[Title/Abstract] OR "traditional tongue diagnosis"[Title/Abstract] OR ((("Tongue"[MeSH Terms] OR "Tongue"[All Fields] OR "tongues"[All Fields] OR "tongue s"[All Fields]) AND ("diagnosable"[All Fields] OR "diagnosi"[All Fields] OR "Diagnosis"[MeSH Terms] OR "Diagnosis"[All Fields] OR "diagnose"[All Fields] OR "diagnosed"[All Fields] OR "Diagnoses"[All Fields] OR "diagnosing"[All Fields] OR "Diagnosis"[MeSH Subheading])) AND "Traditional"[Title/Abstract]) OR ((("Tongue"[MeSH Terms] OR "Tongue"[All Fields] OR "tongues"[All Fields] OR "tongue s"[All Fields]) AND ("diagnosable"[All Fields] OR "diagnosi"[All Fields] OR "Diagnosis"[MeSH Terms] OR "Diagnosis"[All Fields] OR "diagnose"[All Fields] OR "diagnosed"[All Fields] OR "Diagnoses"[All Fields] OR "diagnosing"[All Fields] OR "Diagnosis"[MeSH Subheading])) AND "Traditional"[Title/Abstract]) OR (("tradition"[All Fields] OR "tradition s"[All Fields] OR "Traditional"[All Fields] OR "traditionals"[All Fields] OR "traditions"[All Fields]) AND "tongue diagnoses"[Title/Abstract]) OR (("tradition"[All Fields] OR "tradition s"[All Fields] OR "Traditional"[All Fields] OR "traditionals"[All Fields] OR "traditions"[All Fields]) AND "tongue assessment"[Title/Abstract]) OR (("Tongue"[MeSH Terms] OR "Tongue"[All Fields] OR "tongues"[All Fields] OR "tongue s"[All Fields]) AND "assessment traditional"[Title/Abstract]) OR (("tradition"[All Fields] OR "tradition s"[All Fields] OR "Traditional"[All Fields] OR "traditionals"[All Fields] OR "traditions"[All Fields]) AND "tongue assessments"[Title/Abstract]) OR "Tang"[Title/Abstract] OR "Decoction"[Title/Abstract] OR "Fang"[Title/Abstract] OR "Wan"[Title/Abstract] OR "Keli"[Title/Abstract] OR "San"[Title/Abstract] OR "Zhusheye"[Title/Abstract] OR "Injection"[Title/Abstract] 633,149 20:00:54

4 "Medicine, Chinese Traditional"[Mesh] Most Recent "medicine, chinese traditional"[MeSH Terms] 24,355 19:55:20

3 ("Graves Disease"[Mesh]) OR ((((((((((((((Graves Disease[Title/Abstract]) OR (Disease, Graves[Title/Abstract])) OR (Basedow Disease[Title/Abstract])) OR (Disease, Basedow[Title/Abstract])) OR (Graves' Disease[Title/Abstract])) OR (Disease, Graves'[Title/Abstract])) OR (Exophthalmic Goiter[Title/Abstract])) OR (Exophthalmic Goiters[Title/Abstract])) OR (Goiters, Exophthalmic[Title/Abstract])) OR (Goiter, Exophthalmic[Title/Abstract])) OR (Hyperthyroidism, Autoimmune[Title/Abstract])) OR (Basedow's Disease[Title/Abstract])) OR (Basedows Disease[Title/Abstract])) OR (Disease, Basedow's[Title/Abstract])) "Graves Disease"[MeSH Terms] OR ("Graves Disease"[Title/Abstract] OR "disease graves"[Title/Abstract] OR "basedow disease"[Title/Abstract] OR "disease basedow"[Title/Abstract] OR "Graves Disease"[Title/Abstract] OR "disease graves"[Title/Abstract] OR "exophthalmic goiter"[Title/Abstract] OR "exophthalmic goiters"[Title/Abstract] OR (("Goiter"[MeSH Terms] OR "Goiter"[All Fields] OR "Goiters"[All Fields] OR "goitres"[All Fields] OR "goiterous"[All Fields] OR "goitre"[All Fields]) AND "Exophthalmic"[Title/Abstract]) OR "goiter exophthalmic"[Title/Abstract] OR "hyperthyroidism autoimmune"[Title/Abstract] OR "basedow s disease"[Title/Abstract] OR "basedows disease"[Title/Abstract] OR "disease basedow s"[Title/Abstract]) 23,619 19:53:30

2 (((((((((((((Graves Disease[Title/Abstract]) OR (Disease, Graves[Title/Abstract])) OR (Basedow Disease[Title/Abstract])) OR (Disease, Basedow[Title/Abstract])) OR (Graves' Disease[Title/Abstract])) OR (Disease, Graves'[Title/Abstract])) OR (Exophthalmic Goiter[Title/Abstract])) OR (Exophthalmic Goiters[Title/Abstract])) OR (Goiters, Exophthalmic[Title/Abstract])) OR (Goiter, Exophthalmic[Title/Abstract])) OR (Hyperthyroidism, Autoimmune[Title/Abstract])) OR (Basedow's Disease[Title/Abstract])) OR (Basedows Disease[Title/Abstract])) OR (Disease, Basedow's[Title/Abstract]) "graves disease"[Title/Abstract] OR "disease graves"[Title/Abstract] OR "basedow disease"[Title/Abstract] OR "disease basedow"[Title/Abstract] OR "graves disease"[Title/Abstract] OR "disease graves"[Title/Abstract] OR "exophthalmic goiter"[Title/Abstract] OR "exophthalmic goiters"[Title/Abstract] OR (("Goiter"[MeSH Terms] OR "Goiter"[All Fields] OR "Goiters"[All Fields] OR "goitres"[All Fields] OR "goiterous"[All Fields] OR "goitre"[All Fields]) AND "Exophthalmic"[Title/Abstract]) OR "goiter exophthalmic"[Title/Abstract] OR "hyperthyroidism autoimmune"[Title/Abstract] OR "basedow s disease"[Title/Abstract] OR "basedows disease"[Title/Abstract] OR "disease basedow s"[Title/Abstract] 15,442 19:50:15

1 "Graves Disease"[Mesh] Most Recent "Graves Disease"[MeSH Terms] 18,980 19:46:49

**Cochrane:**

Search Name:

Date Run: 19/12/2023 04:52:01

Comment:

ID Search Hits

#1 MeSH descriptor: [Graves Disease] explode all trees 557

#2 (Graves Disease):ti,ab,kw OR (Disease, Graves):ti,ab,kw OR (Basedow Disease):ti,ab,kw OR (Disease, Basedow):ti,ab,kw OR (Graves' Disease):ti,ab,kw 2691

#3 (Disease, Graves'):ti,ab,kw OR (Exophthalmic Goiter):ti,ab,kw OR (Exophthalmic Goiters):ti,ab,kw OR (Goiters, Exophthalmic):ti,ab,kw OR (Goiter, Exophthalmic):ti,ab,kw 2685

#4 (Hyperthyroidism, Autoimmune):ti,ab,kw OR (Basedow's Disease):ti,ab,kw OR (Basedows Disease):ti,ab,kw OR (Disease, Basedow's):ti,ab,kw 147

#5 #1 OR #2 OR #3 OR #4 2797

#6 MeSH descriptor: [Medicine, Chinese Traditional] explode all trees 1574

#7 (Medicine, Chinese Traditional):ti,ab,kw OR (Traditional Chinese Medicine):ti,ab,kw OR (Chung I Hsueh):ti,ab,kw OR (Hsueh, Chung I):ti,ab,kw OR (Traditional Medicine, Chinese):ti,ab,kw 10468

#8 (Zhong Yi Xue):ti,ab,kw OR (Chinese Traditional Medicine):ti,ab,kw OR (Chinese Medicine, Traditional):ti,ab,kw OR (Traditional Tongue Diagnosis):ti,ab,kw OR (Tongue Diagnoses, Traditional):ti,ab,kw 10486

#9 (Tongue Diagnosis, Traditional):ti,ab,kw OR (Traditional Tongue Diagnoses):ti,ab,kw OR (Traditional Tongue Assessment):ti,ab,kw OR (Tongue Assessment, Traditional):ti,ab,kw OR (Traditional Tongue Assessments):ti,ab,kw 121

#10 (Tang):ti,ab,kw OR (Decoction):ti,ab,kw OR (Fang):ti,ab,kw OR (Wan):ti,ab,kw OR (Keli):ti,ab,kw 7535

#11 (San):ti,ab,kw OR (Zhusheye):ti,ab,kw OR (Injection):ti,ab,kw 95430

#12 #6 OR #7 OR #8 OR #9 OR #10 OR #11 111197

#13 #5 AND #12 347

**Web of science**

权限 # 检索式 数据库 检索结果 运行日期

"

- WOS.IC: 1993 to 2023

- WOS.CCR: 1985 to 2023

- WOS.SCI: 1975 to 2023

- WOS.AHCI: 1975 to 2023

- WOS.BHCI: 2005 to 2023

- WOS.BSCI: 2005 to 2023

- WOS.ESCI: 2018 to 2023

- WOS.ISTP: 1990 to 2023

- WOS.SSCI: 1965 to 2023

- WOS.ISSHP: 1990 to 2023" 1 "TS=(Graves Disease) OR TS=(Disease, Graves) OR TS=(Basedow Disease) OR TS=(Disease, Basedow) OR TS=(Graves' Disease) OR TS=(Disease, Graves') OR TS=(Exophthalmic Goiter) OR TS=(Exophthalmic Goiters) OR TS=(Goiters, Exophthalmic) OR TS=(Goiter, Exophthalmic) OR TS=(Hyperthyroidism, Autoimmune) OR TS=(Basedow's Disease) OR TS=(Basedows Disease) OR TS=(Disease, Basedow's) " Web of Science 核心合集 20522 Tue Dec 19 2023 18:31:03 GMT+0800 (中国标准时间)

"

- WOS.IC: 1993 to 2023

- WOS.CCR: 1985 to 2023

- WOS.SCI: 1975 to 2023

- WOS.AHCI: 1975 to 2023

- WOS.BHCI: 2005 to 2023

- WOS.BSCI: 2005 to 2023

- WOS.ESCI: 2018 to 2023

- WOS.ISTP: 1990 to 2023

- WOS.SSCI: 1965 to 2023

- WOS.ISSHP: 1990 to 2023" 2 "TS=(Medicine, Chinese Traditional) OR TS=(Traditional Chinese Medicine) OR TS=(Chung I Hsueh) OR TS=(Hsueh, Chung I) OR TS=(Traditional Medicine, Chinese) OR TS=(Zhong Yi Xue) OR TS=(Chinese Traditional Medicine) OR TS=(Chinese Medicine, Traditional) OR TS=(Traditional Tongue Diagnosis) OR TS=(Tongue Diagnoses, Traditional) OR TS=(Tongue Diagnosis, Traditional) OR TS=(Traditional Tongue Diagnoses) OR TS=(Traditional Tongue Assessment) OR TS=(Tongue Assessment, Traditional) OR TS=(Traditional Tongue Assessments) OR TS=(Tang) OR TS=(Decoction) OR TS=(Fang) OR TS=(Wan) OR TS=(Keli) OR TS=(San) OR TS=(Zhusheye) OR TS=(Injection) " Web of Science 核心合集 998642 Tue Dec 19 2023 18:42:36 GMT+0800 (中国标准时间)

"

- WOS.IC: 1993 to 2023

- WOS.CCR: 1985 to 2023

- WOS.SCI: 1975 to 2023

- WOS.AHCI: 1975 to 2023

- WOS.BHCI: 2005 to 2023

- WOS.BSCI: 2005 to 2023

- WOS.ESCI: 2018 to 2023

- WOS.ISTP: 1990 to 2023

- WOS.SSCI: 1965 to 2023

- WOS.ISSHP: 1990 to 2023" 3 "#2 AND #1 " Web of Science 核心合集 394 Tue Dec 19 2023 18:46:21 GMT+0800 (中国标准时间)

**Embase:**

No. Query Results Date

#46 #19 AND #45 398 19-Dec-23

#45 #20 OR #21 OR #22 OR #23 OR #24 OR #25 OR #26 OR #27 OR #28 OR #29 OR #30 OR #31 OR #32 OR #34 OR #35 OR #36 OR #37 OR #38 OR #39 OR #40 OR #41 OR #42 OR #43 OR #44 895516 19-Dec-23

#44 'tongue diagnoses, traditional':ab,ti 0 19-Dec-23

#43 'injection':ab,ti 744260 19-Dec-23

#42 'zhusheye':ab,ti 17 19-Dec-23

#41 'san':ab,ti 52934 19-Dec-23

#40 'keli':ab,ti 121 19-Dec-23

#39 'wan':ab,ti 3763 19-Dec-23

#38 'fang':ab,ti 4157 19-Dec-23

#37 'decoction':ab,ti 14042 19-Dec-23

#36 'tang':ab,ti 6286 19-Dec-23

#35 'traditional tongue assessments':ab,ti 0 19-Dec-23

#34 'tongue assessment, traditional':ab,ti 0 19-Dec-23

#32 'traditional tongue assessment':ab,ti 0 19-Dec-23

#31 'traditional tongue diagnoses':ab,ti 0 19-Dec-23

#30 'tongue diagnosis, traditional':ab,ti 0 19-Dec-23

#29 'traditional tongue diagnosis':ab,ti 9 19-Dec-23

#28 'chinese medicine, traditional':ab,ti 47 19-Dec-23

#27 'chinese traditional medicine':ab,ti 1600 19-Dec-23

#26 'zhong yi xue':ab,ti 2 19-Dec-23

#25 'traditional medicine, chinese':ab,ti 8 19-Dec-23

#24 'hsueh, chung i':ab,ti 0 19-Dec-23

#23 'chung i hsueh':ab,ti 0 19-Dec-23

#22 'traditional chinese medicine':ab,ti 41777 19-Dec-23

#21 'medicine, chinese traditional':ab,ti 13 19-Dec-23

#20 'chinese medicine'/exp 76701 19-Dec-23

#19 #3 OR #4 OR #5 OR #6 OR #7 OR #8 OR #9 OR #10 OR #11 OR #12 OR #13 OR #14 OR #15 OR #16 OR #17 29583 19-Dec-23

#17 'disease, basedows':ab,ti 4 19-Dec-23

#16 'basedows disease':ab,ti 4 19-Dec-23

#15 'basedows disease':ab,ti 4 19-Dec-23

#14 'hyperthyroidism, autoimmune':ab,ti 13 19-Dec-23

#13 'goiter, exophthalmic':ab,ti 0 19-Dec-23

#12 'goiters, exophthalmic':ab,ti 0 19-Dec-23

#11 'exophthalmic goiters':ab,ti 1 19-Dec-23

#10 'exophthalmic goiter':ab,ti 69 19-Dec-23

#9 'disease, graves':ab,ti 174 19-Dec-23

#8 'graves disease':ab,ti 17848 19-Dec-23

#7 'disease, basedow':ab,ti 7 19-Dec-23

#6 'basedow disease':ab,ti 436 19-Dec-23

#5 'disease, graves':ab,ti 174 19-Dec-23

#4 'graves disease':ab,ti 17848 19-Dec-23

#3 'graves disease'/exp 26805 19-Dec-23

**Table S2:** League table for efficacy.

| OR 95%CrI | | | | | | |
| --- | --- | --- | --- | --- | --- | --- |
| BLC |  |  |  |  |  |  |
| 1.48 (0.14, 12.6) | HQI |  |  |  |  |  |
| 1.32 (0.24, 6.78) | 0.88 (0.1, 10) | JKLC |  |  |  |  |
| 0.88 (0.12, 5.69) | 0.59 (0.05, 7.83) | 0.67 (0.09, 4.71) | TW |  |  |  |
| 5.32 (1.93, 17.5)a | 3.59 (0.61, 30.85) | 4.05 (1.31, 15.39)a | 6.07 (1.44, 32.6)a | WM |  |  |
| 1.42 (0.21, 8.41) | 0.95 (0.09, 11.85) | 1.08 (0.15, 7.02) | 1.61 (0.19, 13.72) | 0.27 (0.05, 1) | XKCOL |  |
| 0.63 (0.07, 4.27) | 0.42 (0.03, 5.78) | 0.48 (0.05, 3.56) | 0.71 (0.06, 6.72) | 0.12 (0.02, 0.52)a | 0.45 (0.04, 3.94) | YJP |

A means P＜0.05

**Table S3:** League table for TSH.

| MD 95%CrI | | | | | | | | | | | | | |
| --- | --- | --- | --- | --- | --- | --- | --- | --- | --- | --- | --- | --- | --- |
| BLC |  |  |  |  |  |  |  |  |  |  |  |  |  |
| 0.16 (-0.96, 1.28) | HQI |  |  |  |  |  |  |  |  |  |  |  |  |
| -0.04 (-1.16, 1.08) | -0.2 (-1.63, 1.23) | JKLC |  |  |  |  |  |  |  |  |  |  |  |
| -0.65 (-1.8, 0.5) | -0.81 (-2.27, 0.65) | -0.61 (-2.06, 0.85) | JKLT |  |  |  |  |  |  |  |  |  |  |
| -0.02 (-1.23, 1.19) | -0.18 (-1.68, 1.33) | 0.02 (-1.49, 1.53) | 0.63 (-0.9, 2.16) | JSBC |  |  |  |  |  |  |  |  |  |
| 0 (-0.88, 0.87) | -0.15 (-1.4, 1.08) | 0.05 (-1.21, 1.29) | 0.66 (-0.63, 1.93) | 0.03 (-1.31, 1.36) | SJXYD |  |  |  |  |  |  |  |  |
| -0.4 (-1.28, 0.48) | -0.56 (-1.82, 0.69) | -0.36 (-1.62, 0.89) | 0.25 (-1.03, 1.53) | -0.38 (-1.72, 0.96) | -0.41 (-1.45, 0.63) | TW |  |  |  |  |  |  |  |
| 0.21 (-0.27, 0.68) | 0.05 (-0.96, 1.06) | 0.25 (-0.76, 1.26) | 0.86 (-0.19, 1.9) | 0.23 (-0.88, 1.34) | 0.2 (-0.52, 0.94) | 0.61 (-0.12, 1.35) | WM |  |  |  |  |  |  |
| -0.61 (-1.55, 0.36) | -0.77 (-2.06, 0.55) | -0.57 (-1.85, 0.75) | 0.04 (-1.28, 1.39) | -0.59 (-1.96, 0.82) | -0.62 (-1.7, 0.5) | -0.21 (-1.29, 0.91) | -0.82 (-1.63, 0.02) | XHXYP |  |  |  |  |  |
| 0.37 (-0.52, 1.28) | 0.21 (-1.05, 1.48) | 0.41 (-0.84, 1.69) | 1.02 (-0.27, 2.32) | 0.39 (-0.95, 1.75) | 0.37 (-0.68, 1.43) | 0.78 (-0.28, 1.84) | 0.17 (-0.59, 0.93) | 0.99 (-0.16, 2.09) | XHYYP |  |  |  |  |
| -0.72 (-1.61, 0.18) | -0.88 (-2.14, 0.39) | -0.68 (-1.94, 0.6) | -0.07 (-1.36, 1.22) | -0.7 (-2.04, 0.66) | -0.73 (-1.78, 0.33) | -0.32 (-1.37, 0.75) | -0.93 (-1.68, -0.17)a | -0.11 (-1.24, 1) | -1.1 (-2.16, -0.01)a | XKCG |  |  |  |
| -0.24 (-0.91, 0.43) | -0.4 (-1.51, 0.71) | -0.2 (-1.32, 0.92) | 0.41 (-0.74, 1.55) | -0.22 (-1.43, 0.99) | -0.25 (-1.11, 0.63) | 0.16 (-0.71, 1.04) | -0.45 (-0.92, 0.02) | 0.37 (-0.59, 1.31) | -0.62 (-1.51, 0.28) | 0.48 (-0.42, 1.37) | XKCOL |  |  |
| -0.22 (-1.17, 0.68) | -0.38 (-1.68, 0.88) | -0.18 (-1.49, 1.08) | 0.43 (-0.91, 1.71) | -0.19 (-1.59, 1.15) | -0.22 (-1.33, 0.83) | 0.19 (-0.92, 1.24) | -0.43 (-1.24, 0.34) | 0.4 (-0.8, 1.49) | -0.59 (-1.72, 0.48) | 0.5 (-0.63, 1.57) | 0.02 (-0.92, 0.91) | YJP |  |
| -1.11 (-1.97, -0.24)a | -1.26 (-2.5, -0.02)a | -1.06 (-2.31, 0.18) | -0.45 (-1.73, 0.82) | -1.08 (-2.4, 0.24) | -1.11 (-2.14, -0.07)a | -0.7 (-1.73, 0.34) | -1.31 (-2.03, -0.59)a | -0.49 (-1.61, 0.59) | -1.48 (-2.52, -0.43)a | -0.38 (-1.43, 0.66) | -0.86 (-1.73, 0) | -0.89 (-1.93, 0.22) | YKP |

A means P＜0.05

**Table S4**: League table for FT3.

| MD 95%CrI | | | | | | | | | | | | | |
| --- | --- | --- | --- | --- | --- | --- | --- | --- | --- | --- | --- | --- | --- |
| BLC |  |  |  |  |  |  |  |  |  |  |  |  |  |
| 1.73 (-2.46, 5.97) | HQI |  |  |  |  |  |  |  |  |  |  |  |  |
| 2.89 (-1.29, 7.06) | 1.15 (-4.27, 6.58) | JKLC |  |  |  |  |  |  |  |  |  |  |  |
| 1.29 (-1.91, 4.49) | -0.46 (-5.13, 4.26) | -1.6 (-6.26, 3.07) | JKLT |  |  |  |  |  |  |  |  |  |  |
| -0.07 (-4.24, 4.1) | -1.81 (-7.22, 3.57) | -2.96 (-8.37, 2.41) | -1.36 (-6.04, 3.32) | JSBC |  |  |  |  |  |  |  |  |  |
| 0.95 (-2.37, 4.28) | -0.79 (-5.59, 3.99) | -1.94 (-6.7, 2.81) | -0.34 (-4.25, 3.6) | 1.02 (-3.71, 5.79) | SJXYD |  |  |  |  |  |  |  |  |
| 0.77 (-2.41, 3.98) | -0.96 (-5.65, 3.77) | -2.11 (-6.79, 2.58) | -0.51 (-4.31, 3.3) | 0.85 (-3.79, 5.52) | -0.18 (-4.11, 3.77) | TW |  |  |  |  |  |  |  |
| -0.76 (-2.45, 0.95) | -2.5 (-6.35, 1.35) | -3.65 (-7.48, 0.17) | -2.05 (-4.73, 0.65) | -0.69 (-4.49, 3.12) | -1.71 (-4.55, 1.14) | -1.53 (-4.23, 1.16) | WM |  |  |  |  |  |  |
| -0.55 (-3.75, 2.65) | -2.29 (-7, 2.4) | -3.44 (-8.12, 1.24) | -1.84 (-5.67, 1.97) | -0.48 (-5.14, 4.18) | -1.5 (-5.45, 2.41) | -1.33 (-5.16, 2.48) | 0.21 (-2.5, 2.91) | XHXYP |  |  |  |  |  |
| 0 (-4.19, 4.23) | -1.74 (-7.14, 3.72) | -2.88 (-8.29, 2.56) | -1.28 (-5.96, 3.42) | 0.07 (-5.33, 5.48) | -0.94 (-5.72, 3.83) | -0.77 (-5.5, 3.91) | 0.77 (-3.06, 4.61) | 0.56 (-4.13, 5.25) | XHYYP |  |  |  |  |
| 2.93 (-0.34, 6.23) | 1.19 (-3.56, 5.96) | 0.04 (-4.69, 4.76) | 1.64 (-2.25, 5.53) | 2.99 (-1.73, 7.73) | 1.98 (-2.02, 5.97) | 2.15 (-1.76, 6.04) | 3.69 (0.89, 6.49)a | 3.48 (-0.41, 7.39) | 2.92 (-1.83, 7.67) | XKCG |  |  |  |
| 1.32 (-1.13, 3.81) | -0.42 (-4.65, 3.84) | -1.57 (-5.78, 2.65) | 0.03 (-3.18, 3.27) | 1.39 (-2.79, 5.61) | 0.37 (-2.97, 3.75) | 0.54 (-2.68, 3.78) | 2.08 (0.31, 3.87)a | 1.87 (-1.35, 5.11) | 1.31 (-2.9, 5.56) | -1.61 (-4.92, 1.71) | XKCOL |  |  |
| -0.03 (-3.28, 3.23) | -1.77 (-6.52, 3.01) | -2.92 (-7.65, 1.81) | -1.32 (-5.15, 2.58) | 0.05 (-4.64, 4.72) | -0.98 (-4.96, 3.03) | -0.8 (-4.67, 3.04) | 0.73 (-2.04, 3.51) | 0.52 (-3.34, 4.41) | -0.04 (-4.77, 4.71) | -2.95 (-6.9, 1) | -1.35 (-4.64, 1.93) | YJP |  |
| 1.72 (-1.13, 4.61) | -0.02 (-4.47, 4.48) | -1.17 (-5.61, 3.29) | 0.43 (-3.08, 4.01) | 1.79 (-2.64, 6.26) | 0.77 (-2.88, 4.44) | 0.95 (-2.59, 4.51) | 2.48 (0.19, 4.8)a | 2.27 (-1.26, 5.83) | 1.71 (-2.74, 6.19) | -1.21 (-4.84, 2.43) | 0.4 (-2.52, 3.32) | 1.75 (-1.83, 5.37) | YKP |

A means P＜0.05

**Table S5:** League table for FT4.

| MD 95%CrI | | | | | | | | | | | | | |
| --- | --- | --- | --- | --- | --- | --- | --- | --- | --- | --- | --- | --- | --- |
| BLC |  |  |  |  |  |  |  |  |  |  |  |  |  |
| 0.45 (-7.7, 8.58) | HQI |  |  |  |  |  |  |  |  |  |  |  |  |
| 6.65 (-1.5, 14.81) | 6.21 (-4.27, 16.69) | JKLC |  |  |  |  |  |  |  |  |  |  |  |
| 1.64 (-4.71, 8.03) | 1.22 (-8, 10.33) | -5 (-14.16, 4.26) | JKLT |  |  |  |  |  |  |  |  |  |  |
| -1.72 (-10, 6.58) | -2.16 (-12.79, 8.44) | -8.37 (-18.95, 2.28) | -3.35 (-12.69, 5.93) | JSBC |  |  |  |  |  |  |  |  |  |
| 2.93 (-3.48, 9.34) | 2.49 (-6.7, 11.66) | -3.71 (-12.93, 5.5) | 1.3 (-6.41, 8.96) | 4.66 (-4.7, 13.97) | SJXYD |  |  |  |  |  |  |  |  |
| 2.77 (-3.43, 9) | 2.33 (-6.75, 11.41) | -3.87 (-12.99, 5.25) | 1.15 (-6.43, 8.61) | 4.49 (-4.75, 13.69) | -0.18 (-7.72, 7.45) | TW |  |  |  |  |  |  |  |
| -0.86 (-4.23, 2.48) | -1.3 (-8.68, 6.08) | -7.52 (-14.94, -0.06)a | -2.49 (-7.94, 2.91) | 0.86 (-6.72, 8.44) | -3.8 (-9.22, 1.65) | -3.64 (-8.89, 1.61) | WM |  |  |  |  |  |  |
| -0.82 (-7.2, 5.55) | -1.28 (-10.43, 7.87) | -7.47 (-16.71, 1.73) | -2.47 (-10.18, 5.16) | 0.88 (-8.4, 10.17) | -3.78 (-11.44, 3.96) | -3.6 (-11.13, 3.92) | 0.03 (-5.38, 5.45) | XHXYP |  |  |  |  |  |
| 2 (-6.66, 10.7) | 1.55 (-9.32, 12.47) | -4.64 (-15.65, 6.26) | 0.34 (-9.3, 10.01) | 3.7 (-7.3, 14.74) | -0.96 (-10.59, 8.72) | -0.78 (-10.42, 8.81) | 2.85 (-5.14, 10.89) | 2.82 (-6.83, 12.52) | XHYYP |  |  |  |  |
| 6.26 (-0.27, 12.75) | 5.8 (-3.41, 15.09) | -0.39 (-9.69, 8.92) | 4.6 (-3.16, 12.44) | 7.98 (-1.45, 17.42) | 3.32 (-4.47, 11.11) | 3.48 (-4.18, 11.14) | 7.11 (1.54, 12.73)a | 7.09 (-0.68, 14.85) | 4.27 (-5.52, 14.07) | XKCG |  |  |  |
| 3.6 (-1.2, 8.45) | 3.16 (-4.98, 11.34) | -3.04 (-11.22, 5.18) | 1.96 (-4.5, 8.43) | 5.31 (-2.98, 13.64) | 0.67 (-5.82, 7.15) | 0.83 (-5.46, 7.14) | 4.46 (1.02, 7.96)a | 4.43 (-1.99, 10.89) | 1.62 (-7.13, 10.36) | -2.65 (-9.22, 3.96) | XKCOL |  |  |
| 0.55 (-5.78, 6.94) | 0.13 (-9.02, 9.3) | -6.09 (-15.29, 3.16) | -1.08 (-8.78, 6.66) | 2.27 (-7.01, 11.64) | -2.38 (-10.04, 5.35) | -2.21 (-9.69, 5.36) | 1.42 (-3.98, 6.9) | 1.38 (-6.25, 9.1) | -1.44 (-11.09, 8.26) | -5.7 (-13.43, 2.1) | -3.05 (-9.47, 3.43) | YJP |  |
| 4.56 (-1.02, 10.11) | 4.13 (-4.53, 12.73) | -2.08 (-10.73, 6.54) | 2.92 (-4.13, 9.95) | 6.28 (-2.49, 15.03) | 1.62 (-5.42, 8.63) | 1.79 (-5.1, 8.63) | 5.42 (0.96, 9.86)a | 5.4 (-1.63, 12.38) | 2.57 (-6.63, 11.74) | -1.69 (-8.86, 5.41) | 0.96 (-4.72, 6.56) | 4 (-3.05, 10.97) | YKP |

A means P＜0.05

**Table S6:** League table for TGAb.

| MD 95%CrI | | | | | | |
| --- | --- | --- | --- | --- | --- | --- |
| BLC |  |  |  |  |  |  |
| -21.26 (-293.16, 248.4) | HQI |  |  |  |  |  |
| 5.98 (-263.11, 278.19) | 27.37 (-242.76, 299.13) | JKLC |  |  |  |  |
| -25.42 (-217.59, 165.61) | -4.41 (-194.13, 187.32) | -31.37 (-223.28, 158.05) | WM |  |  |  |
| -18.43 (-291.71, 251.88) | 2.53 (-267.47, 274.65) | -24.22 (-295.6, 243.88) | 6.75 (-184.71, 198.48) | XKCG |  |  |
| 57.25 (-164.52, 278.08) | 78.23 (-141.68, 300.3) | 50.79 (-169.93, 270.67) | 82.33 (-27.92, 193.82) | 75.73 (-146.2, 296.64) | XKCOL |  |
| 49.86 (-183.76, 284.19) | 71.28 (-162.34, 306.13) | 44.28 (-191.35, 277.22) | 75.31 (-59.94, 211.09) | 68.21 (-167, 303.33) | -7.16 (-181.17, 167.62) | YKP |

A means P＜0.05

**Table S7:** League table for TPOAb.

| MD 95%CrI | | | | | |
| --- | --- | --- | --- | --- | --- |
| BLC |  |  |  |  |  |
| 4.84 (-91.52, 100.82) | JKLC |  |  |  |  |
| -25.85 (-62.63, 10.5) | -30.62 (-119.65, 58.35) | WM |  |  |  |
| 58 (-42.65, 157.86) | 53.23 (-75.77, 182.09) | 83.82 (-9.67, 177.39) | XKCG |  |  |
| 65.63 (1.89, 129.19)a | 60.87 (-42.63, 164.32) | 91.55 (39.43, 143.89)a | 7.73 (-99.29, 114.7) | XKCOL |  |
| 29.17 (-44.96, 103.06) | 24.41 (-84.93, 134.38) | 54.95 (-9.5, 119.81) | -28.86 (-142.78, 84.86) | -36.56 (-119.77, 46.78) | YKP |

A means P＜0.05

**Table S8:** League table for TRAb.

| MD 95%CrI | | | | | | | | |
| --- | --- | --- | --- | --- | --- | --- | --- | --- |
| BLC |  |  |  |  |  |  |  |  |
| -2.89 (-20.04, 14.12) | JKLT |  |  |  |  |  |  |  |
| -2.17 (-19.38, 14.81) | 0.71 (-21.48, 23.04) | JSBC |  |  |  |  |  |  |
| 2.52 (-10.5, 15.72) | 5.39 (-13.83, 25.13) | 4.67 (-14.59, 24.23) | SJXYD |  |  |  |  |  |
| -5.5 (-12.06, 0.91) | -2.61 (-18.43, 13.24) | -3.32 (-19.09, 12.43) | -8.01 (-19.53, 3.22) | WM |  |  |  |  |
| -4.36 (-17.35, 8.53) | -1.47 (-20.75, 17.87) | -2.14 (-21.56, 17.02) | -6.87 (-23.01, 8.92) | 1.16 (-10.04, 12.35) | XHXYP |  |  |  |
| 2.61 (-15.25, 20.38) | 5.51 (-17.45, 28.34) | 4.82 (-18.12, 27.58) | 0.09 (-20.17, 19.94) | 8.12 (-8.51, 24.65) | 6.97 (-13.01, 26.87) | XKCG |  |  |
| 6.05 (-5.26, 17.44) | 8.95 (-9.36, 27.31) | 8.23 (-9.84, 26.65) | 3.55 (-11.11, 18.19) | 11.57 (2.38, 20.92)a | 10.42 (-3.95, 25.02) | 3.44 (-15.29, 22.54) | XKCOL |  |
| -3.96 (-21.09, 13.16) | -1.07 (-23.57, 21.37) | -1.75 (-24.08, 20.54) | -6.47 (-26.12, 12.83) | 1.54 (-14.27, 17.37) | 0.42 (-19.03, 19.78) | -6.57 (-29.38, 16.28) | -10 (-28.49, 8.14) | YJP |

A means P＜0.05
